# Long-term resting EEG correlates of repetitive mild traumatic brain injury and loss of consciousness: alterations in alpha-beta power

**DOI:** 10.3389/fneur.2023.1241481

**Published:** 2023-08-29

**Authors:** Laura M. Franke, Robert A. Perera, Scott R. Sponheim

**Affiliations:** ^1^Department of Physical Medicine and Rehabilitation, Virginia Commonwealth University, Richmond, VA, United States; ^2^Department of Biostatistics, Virginia Commonwealth University, Richmond, VA, United States; ^3^Minneapolis VA Health Care System, Minneapolis, MN, United States; ^4^Department of Psychiatry and Behavioral Sciences, University of Minnesota, Minneapolis, MN, United States

**Keywords:** mild traumatic brain injury, military, EEG, loss of consciousness, chronic effects, post-traumatic amnesia, cognition

## Abstract

**Objective:**

Long-term changes to EEG spectra after mild traumatic brain injury (mTBI, i.e., concussion) have been reported; however, the role of injury characteristics in long-term EEG changes is unclear. It is also unclear how any chronic EEG changes may underlie either subjective or objective cognitive difficulties, which might help explain the variability in recovery after mTBI.

**Methods:**

This study included resting-state high-density electroencephalography (EEG) and mTBI injury data from 340 service members and veterans collected on average 11 years after injury as well as measures of objective and subjective cognitive functioning. The average absolute power within standard bands was computed across 11 spatial regions of the scalp. To determine how variation in brain function was accounted for by injury characteristics and aspects of cognition, we used regression analyses to investigate how EEG power was predicted by mTBI history characteristics [number, number with post-traumatic amnesia and witnessed loss of consciousness (PTA + LOC), context of injury (combat or non-combat), potentially concussive blast exposures], subjective complaints (TBIQOL General Cognitive and Executive Function Concerns), and cognitive performance (NIH Toolbox Fluid Intelligence and premorbid IQ).

**Results:**

Post-traumatic amnesia (PTA) and loss of consciousness (LOC), poorer cognitive performance, and combat experience were associated with reduced power in beta frequencies. Executive function complaints, lower premorbid IQ, poorer cognitive performance, and higher psychological distress symptoms were associated with greater power of delta frequencies. Multiple regression confirmed the relationship between PTA + LOC, poor cognitive performance, cognitive complaints, and reduced power in beta frequencies and revealed that repetitive mTBI was associated with a higher power in alpha and beta frequencies. By contrast, neither dichotomous classification of the presence and absence of mTBI history nor blast exposures showed a relationship with EEG power variables.

**Conclusion:**

Long-term alterations in resting EEG spectra measures of brain function do not appear to reflect any lasting effect of a history of mTBI or blast exposures. However, power in higher frequencies reflects both injury characteristics and subjective and objective cognitive difficulties, while power in lower frequencies is related to cognitive functions and psychological distress associated with poor long-term outcomes after mTBI.

## 1. Introduction

Resting EEG power spectra are sensitive to mental states and to changes in neural coordination hypothesized to follow mild traumatic brain injury (mTBI) in the chronic phase (1, 2). While EEG spectra have been studied as an evaluative measure of TBI for the past 75 years, much is still unknown concerning the natural history and the clinical significance of spectral changes, as detailed below. These measures were therefore included as exploratory predictors in a prospective cohort study, Long-term Impact of Military Brain Injury Consortium—Chronic Effects of Neurotrauma Consortium (LIMBIC-CENC), designed to assess mid- and long-term outcomes from mTBI and the contributing neurobiological processes.

### 1.1. EEG spectra and mTBI

Early studies of visible alterations in the EEG showed rapid resolution of acute abnormalities after an mTBI, which include power bias toward low frequency and reduction in beta power (3, 4). In mild-to-severe TBI, post-traumatic amnesia (PTA) lasting more than a few hours has been acutely associated with visible EEG abnormalities that are resolved within a longer timeframe of 6 months (5, 6). More severe injuries with a skull fracture and sustained impaired or loss of consciousness (>2 min) also produce a similar diffuse slowing, but to a greater degree (6, 7). Modern quantitative EEG (qEEG) similarly shows increased low-frequency power during PTA also resolving by 6 months postinjury (8). However, the natural history beyond this timeframe is uncertain. Findings of long-term effects (>6 months post-mTBI) have been reported in group studies. Consistently, an increase in low-frequency activity is observed (9–14). Such alterations are reminiscent of the acute changes reported, suggesting a continuity between acute injury and late effects. Enhanced low-frequency oscillations are associated with brain damage, neurodegenerative disease, and loss of or reduced consciousness (15–17) as well as recovery from these states (18). Less consistently, other chronic alterations such as increased gamma activity (19) and reduced alpha and beta coherence (13), and reduced beta power (20) are reported.

While it is clear that mTBI and loss of consciousness (LOC)/PTA especially cause immediate disturbances in the cortical function that typically resolve, it is still unclear whether EEG changes observed in the chronic phase reflect an ongoing, mTBI-specific process. In most studies, it is not clear whether any chronic EEG effects are likely attributable to the mTBI, because of all or some of the following: unvalidated retrospective mTBI classification, small samples, observational design, PCS as study group inclusion criterion, and limited clinical history. mTBI samples can differ significantly from well-matched controls in life experience and functioning (21), and current mental state may confound both EEG and retrospective mTBI classification. However, large samples with carefully detailed and documented history such as LIMBIC-CENC allow the testing of the impact of injury variables, including PTA and LOC, blast exposure, and repetitive injury, which can refine any findings associated with the simple dichotomous classification of mTBI.

### 1.2. EEG spectra and subjective cognitive complaints

While most (~90%) cases are thought to completely resolve, in a small minority of cases complaints persist. However, these complaints are not specific to mTBI–PCS symptoms and are equally observed in non-brain-injured groups with other injuries (22) and military controls (23). Additionally, postconcussive complaints persisting after the acute phase can sometimes be predicted by initial levels of anxiety (24). Yet, there is some indication that subjective cognitive complaints have a neurophysiological basis akin to acute mTBI effects. With routine EEG, abnormal diffuse low-frequency oscillations were observed among a large subset of military airmen with persistent symptoms after TBI, most commonly in those with subdural injuries, but only rarely in those with no symptoms (6). Also shown in that study, slow oscillations tracked the reappearance of symptoms after apparent recovery. With qEEG, similar findings have been reported: increased delta and reduced alpha power with PCS vs. healthy controls 1 year later (12) and acutely declining theta power tracking the PCS symptom abatement 6 weeks later (25). Studies examining the neurophysiologic basis of subjective cognitive complaints in other populations report a similar pattern: higher delta and lower alpha activities differentiate older adults with subjective memory problems from healthy controls (26). Furthermore, the positive relationship between complaints and theta power in EEG (27) and MRI abnormalities (28) is more pronounced in the older adults and TBI groups. In conclusion, it appears that subjective cognitive problems and acute mTBI share a neurophysiology of higher delta–theta power and lower alpha, and this has not been systematically examined in the chronic phase. It is thus not clear to what degree subjective complaints related to or independent of mTBI history are contributing to findings in the chronic phase.

### 1.3. EEG spectra and cognitive ability

A related issue is to what degree mTBI effects lead to objective cognitive problems. Many cohort studies including LIMBIC have examined this, and although not uniform, the results typically indicate no long-term cognitive impairment, on average (29, 30). However, resting EEG indices may track mTBI severity and cognitive function and could indicate a lasting vulnerability, or help explain the persistence of cognitive complaints in some individuals. As might be expected, EEG spectral power is sensitive to general cognitive ability in a myriad of ways. For instance, a large body of studies support that alpha power and theta power are associated with cognitive development, cognitive load, and intelligence (31–33). Others have shown baseline delta and beta power to relate to temporal prediction (34), speed of resting alpha to predict visual attention deficits that in turn strongly predict global cognition (35), and baseline theta to inversely relate to cognitive control (36). Whether chronic changes in EEG after mTBI reflect cognitive dysfunction is not clear.

The present study took advantage of the large sample size and the structured assessment of mTBI history as part of the LIMBIC-CENC study to investigate important predictors of EEG power in chronic mTBI. A second goal was to clarify the extent to which resting oscillatory brain activity was related to subjective complaints and objectively measured cognition in individuals with mTBI.

## 2. Methods

### 2.1. Participants

All participants were enrolled in a large, multi-site, prospective study of long-term outcomes from military mTBI, the LIMBIC-CENC. Participants were all enrolled through either VA or DoD medical facilities. Eligibility criteria were deployment to a post-911 conflict, combat exposure as defined by the Deployment Risk and Resilience Inventory section D (DRRI2) (37) score >1, and 18 years of age or older. Exclusion criteria were any TBI of moderate or higher severity (defined as GCS < 13, loss of consciousness >30 min, post-traumatic amnesia >24 h, or any positive finding on post-injury CT) or major neurologic/neuropsychiatric disorder such as stroke or schizophrenia. More information about the parent study including recruitment is available in prior descriptive publications (38). Eligibility for inclusion in the present analysis was determined by the availability of at least 4 min of artifact-free baseline resting EEG collected from study initiation in 2013 to March 2020. In total, 340 participants met the eligibility criteria. Eligible participants had been enrolled at three different study sites: VA Medical Centers in Richmond, Virginia, and Minneapolis, Minnesota, and DoD site Ft. Belvoir, Virginia.

### 2.2. mTBI assessment

TBI was characterized via validated structured interviews. Trained interviewers conducted the in-person interview, which first assesses all lifetime potential concussive events using a modified version of the Ohio State University TBI Identification (39), and then proceeded with in-depth structured questioning about each event using the VCU Concussion Diagnostic Instrument (40) to determine whether it met the criteria for mTBI as defined by the DoD/VA Clinical Management Guideline (41). Algorithmic mTBI determination was compared with free responses and any corroborating clinical documents to make the final determination. Based on the context of mTBIs incurred, there were five study groups: unexposed, pre-combat mTBI only, combat mTBI only, post-combat mTBI only, and combat and non-combat mTBI. Because of the high demographic and symptom similarity between the groups with combat only and combat plus non-combat, these were combined into one group for the present analysis, as were the pre- and post-combat TBI groups for the same reason; thus, three TBI classification groups resulted: unexposed, combat mTBI, and non-combat mTBI. The interview also generated standardized classifications of injury features for each mTBI: the occurrence of PTA and LOC; whether the LOC was confirmed by a witness; and blast involvement. PTA and LOC with witness corroboration were selected as the primary measure of injury severity, due to the greatest robustness with regard to issues with self-report and memory.

### 2.3. EEG collection and processing

EEG was collected using the Compumedics Neuroscan SynAmpsRT 64 Ag/AgCl channel system at two sites (Richmond/Ft. Belvoir), and the Brain Products ActiChAmp 128 Ag/AgCl channel system at one (Minneapolis), as part of a full day of assessment for the parent study. During recording, EEG was sampled at a rate of 500 Hz (Richmond/Ft. Belvoir) or 1,000 Hz (Minneapolis), and all impedances were kept below 5 kΩ. Participants were instructed to rest quietly with their eyes closed for 10 min, or alternate between 2 min of closing eyes and 2 min of opening eyes, resulting in at least 10 min of eyes-closed EEG. Eyes-open EEG was discarded for this analysis. Participants were monitored to prevent their falling asleep to ensure a common state of relaxed wakefulness. Raw data files were processed using a combination of automated and supervised processing by an investigator (LMF) blinded to all participant information other than the study site. All files were re-referenced to the averaged mastoid channels, DC offset-corrected, and low-pass filtered at 70 Hz using a Hanning window. Bad blocks of large movement artifacts were removed, and bad channels were interpolated with an average of four nearest valid neighbors. Epochs of 4 s (Richmond/Ft. Belvoir) or 1.2 s (Minneapolis) were created, and then any remaining epochs with large amplitude fluctuations (exceeding ±200 uV) were removed. The remaining EEG epochs were each subjected to FFT with a Hanning window with a width of 10%, and then, the results were averaged to produce average spectra for each channel for the entire recording period. The multichannel data were averaged to create regional averages within standard power bands: delta (1–4 Hz), theta (4–8 Hz), alpha (8–12.5 Hz), beta (12.5–35.0 Hz), and gamma (35.0–70 Hz). Because the 128-channel system used is based on the same 10–20 standard as implemented in the 64-channel system, the same channel landmarks could be used to define the scalp regions: anterior of FCz = anterior; posterior of CPz = posterior; remaining central region between FCz and CPz, inclusive of these=central; midline electrodes (^*^z) = midline; left of midline = left; right of midline = right. For temporal regions, landmarks were as follows: lateral of C5 = left temporal; lateral of C6 = right temporal.

### 2.4. Cognitive functioning and other measures

Participants completed self-administered questionnaires to measure subjective cognitive functioning after TBI: TBIQOL General Cognitive Concerns (42) and psychological symptoms of depression via PHQ-9 (43), and PTSD via PCL-5 (44). Participants' military status, pay group, combat duty history, and service-connected disability were self-reported. Cognitive performance was measured using the validated NIH Toolbox Fluid Intelligence Measure, a combination of performance on several tests of fluid ability (processing speed, efficiency, and working memory): the Dimensional Change Card Sort test of executive function, the Flanker inhibitory control and attention Test, a Picture Sequence memory test, a List Sorting working memory test, and a Pattern Comparison processing speed test (45). Premorbid intellectual ability was assessed using the Test of Premorbid Function (TOPF) (46).

### 2.5. Statistical methods and analysis

Descriptive statistics for the overall group and TBI subgroups were produced, and group differences were evaluated using independent *t*-tests for continuous variables or chi-square tests for categorical variables. EEG effects were evaluated with unadjusted (simple) linear single-step regression and adjusted (multiple) single-step linear regression models for each of the 11 regions and 5 bands. Before conducting regressions, it was verified that EEG power by band did not vary by data collection site, and all hypothesized predictors were verified to have a VIF < 5 to prevent issues due to multicollinearity. Time since injury/index date was removed due to VIF > 5; only age was included as a demographic predictor due to known effects of age on EEG and small numbers of women in the sample. Predictor correlations are given in Table 1. The final set of predictors included in the regression models were as follows: DRRI2 combat experience, TBIQOL Cognitive Concerns, TBIQOL Executive Function, NIH Toolbox Fluid Cognition Composite, TOPF, Age, PCL-5, PHQ-9, TBI (unexposed; exposed combat; and exposed non-combat), number of blast potential concussive events (PCEs), number of mTBIs, number of mTBIs with witnessed LOC and PTA). Statistical significance was determined using an alpha of 0.05 adjusted by the Benjamini–Hochberg method (47) to control the false discovery rate for each family of tests defined by EEG band and regression type (simple or multiple); for example, all simple regressions of delta band constituted a family.

**Table 1:** Predictor correlations.

	**DRRI2**	**TBIQOL cognition**	**TBIQOL executive function**	**Fluid cognition**	**TOPF**	**Age in years**	**PCL-5**	**PHQ-9**	**Number of blast PCEs**	**Number of positive mTBIs**	**Number mTBIs with PTA and witnessed LOC**
DRRI2	1										
TBIQOL cognition	−0.32	1									
TBIQOL executive function	−0.23	0.84	1								
Fluid cognition	−0.07	0.27	0.27	1							
TOPF	−0.01	0.12	0.18	0.33	1						
Age in years	−0.2	0.05	0.03	−0.41	−0.09	1					
PCL-5	0.31	−0.65	−0.67	−0.35	−0.27	0.03	1				
PHQ-9	0.26	−0.7	−0.72	−0.3	−0.23	−0.02	0.84	1			
Number of blast PCEs	0.5	−0.19	−0.12	0	0.05	−0.07	0.1	0.1	1		
Number of positive mTBIs	0.31	−0.25	−0.24	−0.09	0.01	0	0.18	0.18	0.34	1	
Number mTBIs with PTA and witnessed LOC	0.18	−0.11	−0.15	−0.15	−0.09	−0.01	0.19	0.18	0.15	0.42	1

Predictor correlations were computed as regression model diagnostics and not for focal hypothesis testing; thus, no *p*-values were assessed for significance.

DRRI, Deployment Risk and Resilience Inventory; TBIQOL, TBI Quality of Life inventory; PCL-5, PTSD Checklist 5; PHQ-9, Patient Health Questionnaire-9; PCE, potential concussive event; PTA, post-traumatic amnesia; LOC, loss of consciousness.

## 3. Results

Demographic information for the entire sample and the key TBI study groups is presented in Table 2. While similar in age and other demographic characteristics, groups differed in terms of combat experience and psychological functioning. Both TBI groups reported higher psychological and cognitive complaints. Psychoactive-CNS medication information for the sample is presented in Table 3. While medication use, especially serotonin modulators, was very common, there was no difference in usage rates between the study groups.

**Table 2:** Demographic and descriptive statistics.

	**Overall**	**Unexposed**	**Combat mTBI**	**Non-combat TBI**	***p*-value**
*n*	328	82	151	95	
DRRI2 combat experience [mean (SD)]	34.84 (14.63)	27.90 (9.21)	42.28 (15.32)	28.99 (11.46)	< 0.001
**Currently in the military?**					
Yes (%)	63 (19.2)	11 (13.4)	38 (25.2)	14 (14.7)	0.04
**Combat role (%)**					
Combat	77 (23.5)	14 (17.1)	49 (32.5)	14 (14.7)	0.04
Combat service support	80 (24.4)	20 (24.4)	33 (21.9)	27 (28.4)	
Combat support	150 (45.7)	43 (52.4)	60 (39.7)	47 (49.5)	
Other	21 (6.4)	5 (6.1)	9 (6.0)	7 (7.4)	
**Pay group (%)**					
No response/missing	1 (0.3)	0 (0.0)	1 (0.7)	0 (0.0)	0.318
Enlisted	258 (78.7)	63 (76.8)	125 (82.8)	70 (73.7)	
Officer	69 (21.0)	19 (23.2)	25 (16.6)	25 (26.3)	
Most recent pay grade[mean (SD)]	5.61 (1.61)	5.44 (1.48)	5.86 (1.59)	5.43 (1.72)	0.108
**Service branch (%)**					
No response/missing	1 (0.3)	0 (0.0)	1 (0.7)	0 (0.0)	0.435
Air force	24 (7.3)	8 (9.8)	8 (5.3)	8 (8.4)	
Army	246 (75.0)	62 (75.6)	115 (76.2)	69 (72.6)	
Marine corps	35 (10.7)	4 (4.9)	19 (12.6)	12 (12.6)	
Navy	22 (6.7)	8 (9.8)	8 (5.3)	6 (6.3)	
**Service-connected disability (%)**					
No response/don't know	2 (0.6)	0 (0.0)	1 (0.7)	1 (1.1)	0.073
N/A	63 (19.2)	11 (13.4)	38 (25.2)	14 (14.7)	
No	34 (10.4)	11 (13.4)	9 (6.0)	14 (14.7)	
Yes	229 (69.8)	60 (73.2)	103 (68.2)	66 (69.5)	
**Service-connected disability (%)**					
N/A	104 (31.7)	22 (26.8)	52 (34.4)	30 (31.6)	0.005
0%	1 (0.3)	1 (1.2)	0 (0.0)	0 (0.0)	
10%	15 (4.6)	7 (8.5)	0 (0.0)	8 (8.4)	
20%	9 (2.7)	3 (3.7)	1 (0.7)	5 (5.3)	
30%	13 (4.0)	9 (11.0)	2 (1.3)	2 (2.1)	
40%	15 (4.6)	3 (3.7)	7 (4.6)	5 (5.3)	
50%	14 (4.3)	3 (3.7)	6 (4.0)	5 (5.3)	
60%	30 (9.1)	7 (8.5)	14 (9.3)	9 (9.5)	
70%	24 (7.3)	7 (8.5)	10 (6.6)	7 (7.4)	
80%	23 (7.0)	4 (4.9)	13 (8.6)	6 (6.3)	
90%	33 (10.1)	9 (11.0)	16 (10.6)	8 (8.4)	
100%	47 (14.3)	7 (8.5)	30 (19.9)	10 (10.5)	
TBIQOL cognitive concerns [mean (SD)]	33.38 (10.33)	37.44 (9.64)	30.39 (10.36)	34.65 (9.45)	< 0.001
TBIQOL executive function [mean (SD)]	37.76 (8.27)	40.46 (7.30)	35.59 (8.40)	38.89 (8.03)	< 0.001
Fluid cognition [mean (SD)]	99.23 (12.70)	101.02 (12.96)	98.04 (12.32)	99.52 (13.00)	0.236
TOPF [mean (SD)]	42.95 (11.97)	42.88 (12.04)	42.42 (12.04)	43.87 (11.88)	0.655
Sex = male (%)	286 (87.2)	70 (85.4)	138 (91.4)	78 (82.1)	0.089
Age in years [mean (SD)]	43.65 (9.99)	43.99 (10.56)	42.65 (9.82)	44.95 (9.70)	0.201
**Education (%)**					
Grade 12 or GED (high school graduate)	39 (11.9)	11 (13.4)	22 (14.6)	6 (6.3)	0.174
College 1 year to 3 years (some college or technical school)	112 (34.1)	28 (34.1)	55 (36.4)	29 (30.5)	
College 4 years or more (college graduate)	177 (54.0)	43 (52.4)	74 (49.0)	60 (63.2)	
Ethnicity = not Hispanic or Latino (%)	304 (93.5)	78 (95.1)	138 (92.6)	88 (93.6)	0.759
**Marital status (%)**					
Never married	47 (17.6)	16 (22.5)	18 (15.7)	13 (16.0)	0.443
Married	220 (82.4)	55 (77.5)	97 (84.3)	68 (84.0)	
Divorced	0 (0.0)	0 (0.0)	0 (0.0)	0 (0.0)	
Race = white (%)	222 (67.7)	51 (62.2)	105 (69.5)	66 (69.5)	0.471
PCL-5 [mean (SD)]	23.96 (19.79)	17.16 (17.45)	30.23 (20.16)	19.87 (18.23)	< 0.001
PHQ-9 [mean (SD)]	7.32 (6.25)	5.42 (5.03)	8.65 (6.36)	6.83 (6.59)	0.001
Number of PCEs [mean (SD)]	5.26 (2.85)	3.44 (2.16)	6.34 (2.93)	5.11 (2.40)	< 0.001
Number of blast PCEs [mean (SD)]	3.19 (2.21)	2.51 (1.86)	3.82 (2.40)	2.77 (1.90)	< 0.001
Time since the injury or index date in years [mean (SD)]	10.71 (5.85)	11.69 (6.74)	10.45 (5.18)	10.30 (6.00)	0.215
Number of mTBIs [mean (SD)]	1.82 (1.81)	0.00 (0.00)	2.85 (1.76)	1.76 (1.37)	< 0.001
Number mTBIs with PTA and LOC [mean (SD)]	0.33 (0.61)	0.00 (0.00)	0.54 (0.74)	0.27 (0.51)	< 0.001

*p*-values are the result of ANOVA with factor of group, or chi-square test with group, as appropriate.

DRRI, Deployment Risk and Resilience Inventory; TBIQOL, TBI, Quality of Life inventory; PCL-5, PTSD Checklist 5; PHQ-9, Patient Health Questionnaire-9; PCE, potential concussive event.

**Table 3:** Medications by study group.

	**Unexposed**	**Combat mTBI**	**Non-combat TBI**	***p*-value**
*n*	82	151	95	
SSRI (%)	15 (51.7)	43 (53.1)	21 (47.7)	0.848
Atypical antipsychotic (%)	6 (20.7)	13 (16.0)	2 (4.5)	0.095
Opioid (%)	11 (37.9)	21 (25.9)	10 (22.7)	0.334
Adrenergic antagonist (%)	1 (3.4)	5 (6.2)	2 (4.5)	0.829
Barbituate-stimulant analgesic (%)	1 (3.4)	7 (8.6)	2 (4.5)	0.513
Hypnotic (non-benzodiazepine; %)	3 (10.3)	10 (12.3)	5 (11.4)	0.956
Serotonin agonist (%)	3 (10.3)	15 (18.5)	6 (13.6)	0.532
SNRI (%)	1 (3.4)	8 (9.9)	5 (11.4)	0.484
Antiepileptic (%)	5 (17.2)	25 (30.9)	9 (20.5)	0.238
Tricyclic antidepressant (%)	1 (3.4)	5 (6.2)	3 (6.8)	0.821
Anxiolytic (non-benzodiazepine; %)	3 (10.3)	10 (12.3)	5 (11.4)	0.956
Benzodiazepine (%)	2 (6.9)	12 (14.8)	4 (9.1)	0.427
Stimulant (%)	0 (0.0)	5 (6.2)	5 (11.4)	0.154
Caffeine (%)	1 (3.4)	5 (6.2)	2 (4.5)	0.829
Adrenergic agonist (%)	1 (3.4)	4 (4.9)	3 (6.8)	0.808

Percentages reported are based on the number of medications reported. *p*-values are the result of ANOVA of the group factor.

SSRI, selective serotonin reuptake inhibitor; SNRI, selective norepinephrine reuptake inhibitor.

### 3.1. Unadjusted analysis

There was no statistically significant effect of TBI classification (unexposed vs. combat vs. non-combat) or the number of blast PCEs on EEG power. However, significantly increased delta power accompanied higher scores on the PHQ-9 (unstandardized coefficient range across all electrode locations 0.01–0.036, all *p*-values of < 0.01) and PCL-5 (coefficient range 0.01–0.06, all *p*-values of < 0.001), higher levels of executive cognitive complaints (coefficient range −0.02 to −0.07, all *p*-values of < 0.02), and poorer current (coefficient range −0.02 to −0.08, all *p*-values of < 0.01), and premorbid cognitive function (coefficient range −0.02 to −0.08, all *p*-values of < 0.01). Furthermore, significantly reduced beta power accompanied more mTBIs with PTA+LOC (coefficient range −0.01 to −0.09, all *p*-values of < 0.003), and higher levels of combat experience (coefficient range −0.002 to −0.003, all *p*-values of < 0.003). Full regression parameters for models with significant effects after FDR alpha adjustment are shown in Table 4. All regression models are available in the Supplementary material.

**Table 4:** Regression models for each scalp region and power band with significant effect after alpha correction for multiple comparisons.

**Measure**	**Location**	**Unadjusted**				**Adjusted**				
		**Est**.	**SE**	***t***	***p*** **-value**	**Est**.	**SE**	***t***	β	***p*** **-value**
**A. Delta band**										
Fluid cognition	Anterior R	−0.071	0.015	−4.591	0.000	−0.069	0.019	−3.666	−0.250	0.000
	Anterior mid	−0.083	0.018	−4.575	0.000					
	Anterior L	−0.065	0.015	−4.346	0.000	−0.062	0.018	−3.467	−0.235	0.001
	Central R	−0.036	0.010	−3.797	0.000					
	Central L	−0.035	0.010	−3.630	0.000					
	Central mid	−0.053	0.015	−3.462	0.001					
	Temporal R	−0.020	0.007	−3.102	0.002					
	Posterior mid	−0.020	0.007	−2.759	0.006					
PCL-5	Central R	0.032	0.006	5.338	0.000	0.043	0.012	3.553	0.392	0.000
	Central L	0.031	0.006	5.189	0.000	0.040	0.012	3.294	0.364	0.001
	Anterior mid	0.058	0.011	5.159	0.000					
	Posterior R	0.019	0.004	5.090	0.000	0.033	0.008	4.291	0.477	0.000
	Central mid	0.047	0.009	5.051	0.000	0.040	0.012	3.294	0.352	0.002
	Posterior mid	0.026	0.004	5.813	0.000	0.043	0.009	4.613	0.508	0.000
	Posterior L	0.018	0.004	4.798	0.000	0.036	0.008	4.705	0.520	0.000
	Anterior L	0.038	0.009	4.130	0.000					
	Anterior R	0.039	0.010	4.093	0.000					
	Temporal R	0.016	0.004	3.816	0.000					
	Temporal L	0.013	0.004	3.533	0.000					
PHQ-9	Anterior mid	0.125	0.036	3.460	0.001					
	Posterior mid	0.047	0.015	3.221	0.001					
	Central R	0.058	0.019	3.016	0.003					
	Central L	0.058	0.019	3.009	0.003					
	Central mid	0.091	0.030	2.993	0.003					
	Anterior L	0.089	0.030	2.965	0.003					
	Anterior R	0.091	0.031	2.937	0.004					
	Posterior R	0.033	0.012	2.746	0.006					
Executive function TBIQOL	Posterior mid	−0.033	0.011	−2.932	0.004					
	Anterior mid	−0.076	0.027	−2.824	0.005					
	Central R	−0.038	0.014	−2.638	0.009					
	Central L	−0.037	0.014	−2.606	0.010					
	Posterior R	−0.023	0.009	−2.519	0.012					
	Anterior L	−0.054	0.022	−2.486	0.013					
TOPF	Anterior mid	−0.083	0.019	−4.410	0.000					
	Central R	−0.044	0.010	−4.379	0.000					
	Central mid	−0.066	0.016	−4.155	0.000					
	Central L	−0.042	0.010	−4.133	0.000					
	Anterior L	−0.060	0.016	−3.846	0.000					
	Anterior R	−0.059	0.016	−3.669	0.000					
	Posterior L	−0.019	0.006	−2.965	0.003					
	Posterior mid	−0.022	0.008	−2.871	0.004					
	Temporal R	−0.020	0.007	−2.836	0.005					
	Posterior R	−0.017	0.006	−2.739	0.007					
**B. Alpha band**										
Number mTBIs	Posterior R					0.396	0.137	2.896	0.227	0.004
PCL-5	Central mid					0.038	0.013	2.985	0.373	0.001
	Posterior mid					0.059	0.018	3.250	0.362	0.001
	Anterior mid					0.049	0.016	3.066	0.353	0.002
	Central R					0.038	0.013	2.985	0.342	0.003
	Central L					0.037	0.012	2.953	0.339	0.003
	Anterior L					0.036	0.012	2.909	0.335	0.004
	Anterior R					0.036	0.013	2.770	0.320	0.006
PHQ-9	Posterior mid					−0.214	0.059	−3.621	−0.414	0.000
	Posterior L					−0.175	0.052	−3.359	−0.386	0.001
	Central mid					−0.133	0.041	−3.228	−0.394	0.001
	Temporal R					−0.071	0.021	−3.338	−0.390	0.001
	Central R					−0.133	0.041	−3.228	−0.380	0.001
	Temporal L					−0.064	0.020	−3.203	−0.377	0.002
	Central L					−0.127	0.040	−3.151	−0.371	0.002
	Anterior mid					−0.154	0.051	−3.005	−0.355	0.003
	Anterior L					−0.119	0.040	−2.982	−0.352	0.003
	Anterior R					−0.123	0.042	−2.930	−0.347	0.004
**C. Beta band**										
DRRI2	Posterior L	−0.003	0.001	−3.235	0.001					
	Posterior mid	−0.003	0.001	−3.106	0.002					
	Posterior R	−0.002	0.001	−3.078	0.002					
	Temporal L					−0.003	0.001	−2.823	−0.204	0.005
Fluid cognition	Posterior mid	0.004	0.001	4.281	0.000	0.004	0.001	3.425	0.229	0.001
	Posterior R	0.004	0.001	3.891	0.000	0.003	0.001	2.893	0.196	0.004
	Posterior L	0.004	0.001	3.809	0.000	0.003	0.001	2.782	0.187	0.006
	Temporal L					0.003	0.001	2.847	0.190	0.005
Number mTBIs	Temporal R					0.027	0.009	3.103	0.245	0.002
	Central R					0.028	0.010	2.780	0.216	0.006
	Temporal L					0.024	0.009	2.767	0.212	0.006
Number PTA +	Central L	−0.085	0.021	−4.042	0.000	−0.107	0.025	−4.231	−0.263	0.000
LOC	Posterior mid	−0.076	0.019	−3.914	0.000	−0.075	0.023	−3.219	−0.197	0.001
	Posterior L	−0.006	0.007	−0.844	0.000	−0.083	0.024	−3.476	−0.214	0.001
	Central R	−0.080	0.021	−3.712	0.000	−0.100	0.025	−3.987	−0.248	0.000
	Posterior R	−0.070	0.019	−3.682	0.000	−0.072	0.023	−3.102	−0.193	0.002
	Central mid	−0.085	0.023	−3.638	0.000	−0.089	0.027	−3.355	−0.212	0.001
	Temporal L	−0.065	0.019	−3.434	0.001	−0.088	0.022	−4.049	−0.249	0.000
	Anterior L	−0.068	0.020	−3.341	0.001	−0.082	0.025	−3.318	−0.211	0.001
	Anterior mid	−0.072	0.024	−3.058	0.002	−0.079	0.028	−2.828	−0.182	0.005
	Temporal R					−0.068	0.022	−3.115	−0.197	0.002
Cognitive concerns TBIQOL	Temporal L					−0.006	0.002	−2.840	−0.308	0.005

Regression parameters for the EEG bands and locations with significant effects after alpha correction to control false discovery rate at 5%. For clarity, models with non-significant effects are not included (please see Supplementary material). No effects were observed in the theta or gamma band.

Est, non-standardized regression coefficient estimate; SE, standard error; β, standardized regression coefficient.

### 3.2. Adjusted analysis

**Figure 1:**
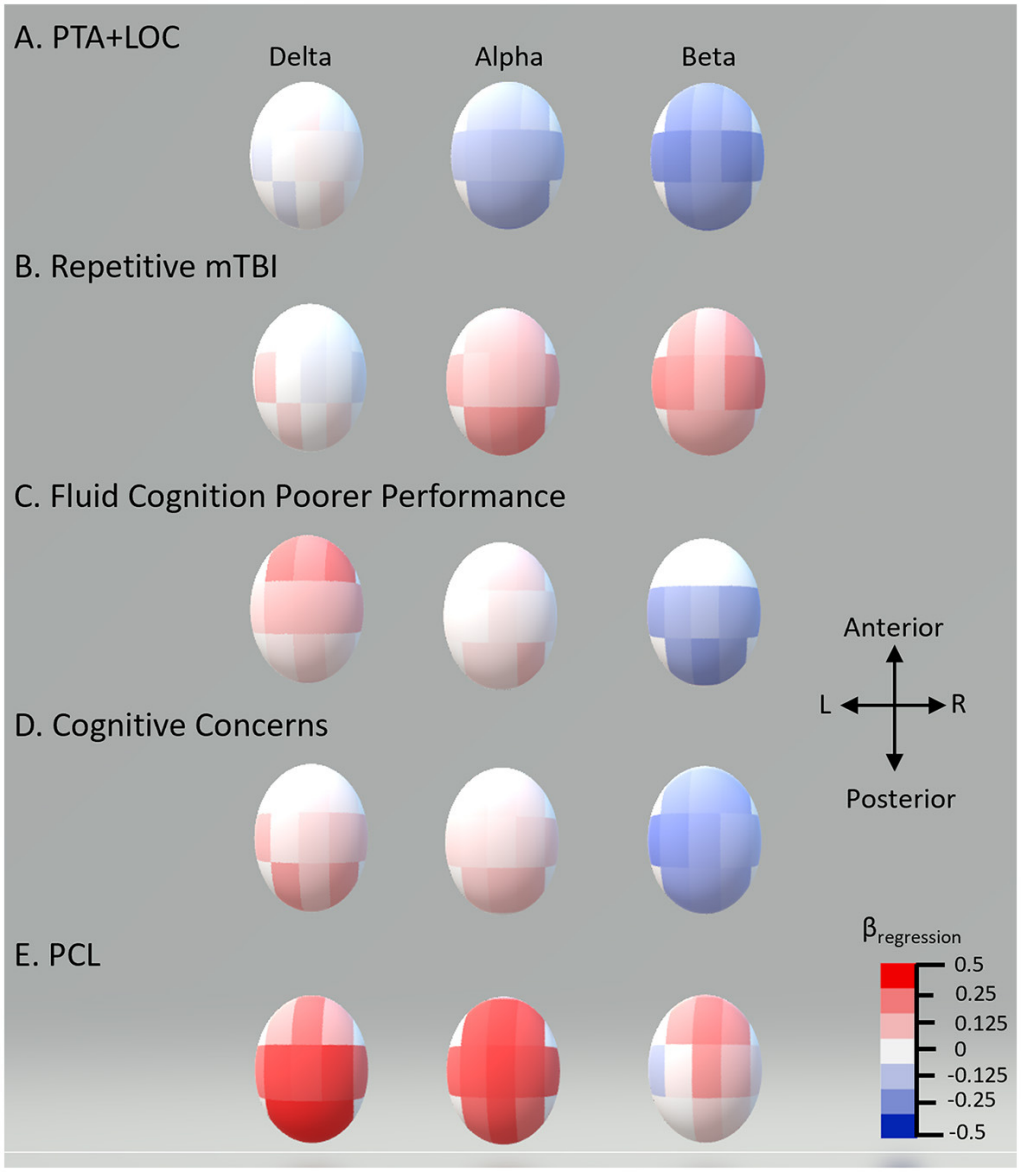
Standardized multiple regression coefficients (β) by scalp recording region. Topographic plot of standardized multiple regression coefficients for each of the 11 regions for the EEG bands showing significant differences in multiple regression.

In the adjusted analysis, there was no statistically significant effect of TBI classification (unexposed vs. combat vs. non-combat) or the number of blast PCEs on EEG power. Delta power was found to increase for persons with poorer current cognitive function (unstandardized coefficient range −0.06 to −0.07, all *p*-values of < 0.002) and greater PCL-5 symptoms (coefficient range 0.03–0.04, all *p*-values of < 0.003). Alpha power was found to be higher with an increasing number of mTBIs (coefficient 0.396, *p* = 0.004) and PCL-5 severity (coefficient range 0.04–0.06, all *p*-values of < 0.003). Alpha was lower with PHQ-9 severity (coefficient range −0.06 to −0.2, all *p*-values of < 0.005). Beta power was reduced with higher PTA + LOC (coefficient range −0.07 to −0.1, all *p*-values of < 0.006), cognitive concerns (coefficient −0.006, *p* = 0.005), and combat experience (coefficient −0.003, *p* = 0.005), and with poorer cognitive function (coefficient range 0.003–0.004, all *p*-values of < 0.007). Beta was higher with an increasing number of mTBIs (coefficient range 0.02–0.03, all *p*-values of < 0.007). Standardized effect sizes ranged from 0.2 to 0.5, in the small to moderate range. Significant effects after FDR correction with standardized coefficients are shown in Table 4. The topographies of adjusted effects are shown in Figure 1.

### 3.3. Sensitivity analyses

Sensitivity analyses were conducted *post-hoc* to assess the impact of differences across site/EEG system and collection procedures and the effect of PTA exposures without LOC. For the site, mixed-effects models confirmed zero or near zero variance for the site random effect, indicating no impact of the site on outcomes. For the number of mTBI with PTA in those without LOC, there was no relationship with EEG outcomes in either simple or multiple regression, and multiple regression effects of the original set of predictors were not meaningfully changed with the addition of the PTA but no LOC variable.

## 4. Discussion

We found that resting EEG power in the chronic phase of mTBI was not affected by simple mTBI history, regardless of combat context or blast exposures. Instead, higher frequency (alpha–beta) oscillations were related to mTBI dose-severity variables, as well as cognitive performance. Low-frequency oscillations were related to distress symptoms and current cognitive functioning accounting for premorbid IQ.

### 4.1. Traumatic brain injury features are related to alpha–beta changes

The central finding of this analysis was that two injury features were independently associated with chronic changes in the alpha and beta bands. Alpha activity and beta activity are putatively involved in the top-down modulation of sensorimotor processing (48) and control of attention (49). PTA + LOC was associated with a widespread reduction in beta power, even after accounting for differences in other contributors, including the current fluid cognitive ability. This finding extends to the chronic phase observations of beta reduction in acute mTBI (4) and subacute mTBI (20), as well as with moderate–severe injury with attentional deficits (50) and beta coherence reduction in chronic mTBI (13). Similarly, subacute PTA with object feature binding dysfunction exhibited a bias away from middle-frequency power toward low frequency (8). Together with the present result, these provide convergent evidence that acute disruptions underlying observable disturbances of orientation and consciousness can persist, especially with repeated exposure. Presently, the reduction in beta power was also independently associated with poorer fluid cognition and cognitive concerns and so is consistent with a chronic neurophysiologic alteration that can produce true cognitive dysfunction and accurate complaints. The beta reduction may indicate reduced activity in the self-referential resting-state network and/or more activity in the sensory areas, especially auditory (51), and a lower level of attentional engagement and focus (52, 53).

Repetitive mTBI, after taking into account symptom level and PTA + LOC, was associated with higher resting posterior alpha. Higher posterior alpha is generally found with deactivation of the posterior cortex, especially visual, and a lower sampling rate of the focus of attention. Higher resting alpha predicted longer attentional blink (52), global perceptual bias (54), and increased susceptibility to interference in a Flanker task (53). High resting alpha is negatively related to sensory cortices rCBF, especially visual (51). Repetitive mTBI was also associated with a higher beta, indicating that multiple mTBIs may result in an abnormal imbalance of activity in the alpha–beta bands, i.e., in the alpha–beta ratio. Previous research would suggest an imbalance would accompany attentional effects that are more subtle than detectable by standard neuropsychological assessment: the alpha/beta ratio was related to the scope of attention in time and space (52, 54).

In summary, PTA + LOC exposures and repetitive mTBI appear to have different long-term effects on the resting alpha–beta activity previously related to attention and selection. This finding was somewhat unexpected because both repetitive mTBI and LOC are considered features, which may increase the risk for poor outcomes. However, these two injury variables thus should be considered independent risk factors, with different chronic effects as presently observed. Alpha–beta resting activity should be a focus of future research into chronic mTBI biomarkers; notably, recent findings convergently highlight these two bands as a discriminant for mTBI vs. PTSD (55). Beta power reduction in particular may be a useful biomarker because of its strong relationship with PTA + LOC, with and without statistical controls, and with objective cognitive performance. Furthermore, the reduction of beta power has convergent findings in similar populations as detailed above.

### 4.2. Cognitive function is related to delta and beta activity

A higher level of delta and reduced beta was predicted by poorer fluid cognitive ability. No effect of current cognition or premorbid IQ was observed in alpha or theta. This may be due to the relatively small age distribution of the present sample or the limited nature of the cognitive tests (we did not evaluate full-scale IQ). The lack of theta effects may be due to the choice of fluid cognition as the cognitive domain of interest, which emphasizes processing speed, short-term memory, and executive processing, and very little long-term memory or vigilance demand associated with theta activity (56, 57). The involvement of delta and beta, however, is consonant with fluid cognition demands. Delta and delta–beta coordination are correlated with integrating cognitive functions over large areas of the brain (58), processes, for instance, underlying P300 (59). Delta oscillations have been implicated in response inhibition and balance between internal and external representations (60), while prestimulus delta–beta coordination underlies auditory temporal prediction accuracy (34) and cortical excitability for movement (61), and delta coordinates higher frequency activity to direct attention (62). Taken in the context of the findings of the present study, the delta–beta system underlying fluid cognition is relevant to late chronic mTBI, especially beta, shown similarly in moderate–severe injury by Shah et al. (50).

### 4.3. Subjective distress associated with delta elevation

Greater distress on all symptom measures was related to higher delta power. This was similar to effects reported in the acute phase of mTBI and other populations with subjective complaints, especially the higher power in low frequencies. Therefore, there appears to be a continuity between the slow wave correlate of symptoms early and much later (years) in recovery from injury. Furthermore, the symptoms continued to predict greater delta activity even after controlling for cognitive function, suggesting the slow oscillations track sensitivity to or expression of perceived difficulties, in addition to the cognitive processes described earlier. This illustrates a deep modulatory role of the delta networks and is interesting in light of the characterization of delta oscillations as critical in motivation, mood, and appetitive states (63), as well as biasing toward internal representations (64).

Finally, while previous studies have reported acute and chronic slowing with mTBI, there was no significant effect of any of the injury variables in the present study. This may be because of the long time since injury (10 years on average in the present study compared with 9 months for Franke et al.). However, the increased slow waves also represented a state of reduced cognition above and beyond the distress. Therefore, there appears to be a true dysfunctional state characterized by increased delta, but it is not related to the injury, at least at long lags. Therefore, there may be a risk of misattribution and bias of positive retrospective mTBI classification when cognitive problems and distress symptoms are emphasized and when the phase is very chronic (2+ years out from injury).

### 4.4. Evolution of mTBI effects on EEG over time

Previous analyses showed that PTA affected the delta band (8, 10), but this was relatively early after injury, in the subacute phase or on average less than a year after worst blast exposure. However, the present analysis suggests that very chronic impacts are on the alpha–beta bands, a finding consistent with the report of Lewine et al. (13). Together, these findings paint a picture of effects evolving over a very long time period, beyond just the 3 months typically associated with the resolution of symptoms and neuropsychological deficits. The pattern is acute widespread impacts to delta, theta, alpha, and beta. Then, during the subacute-early chronic phase, TBI effects are still observable in delta. Finally, in the very chronic phase, delta effects are primarily attributable to “internalizing symptoms” with subtle effects in alpha–beta for the higher dose (more and more severe) of mTBI. This transition from widespread effects including delta to alpha–beta suggests an evolution of neurophysiological effects of mTBI with PTA/LOC from deep modulatory (delta) to altered attention and sensory filtering (alpha–beta); the injury gets “better” in the sense of less extensive neurophysiological effects but still affects higher order processes. This hypothesized trajectory will continue to be tracked in the longitudinal LIMBIC-CENC analyses.

## 5. Limitations

The limitations of the present study include, foremostly, an observational design. Because of unmeasured variables and cohort effects in this type of design, one can never truly infer that the injury features are the cause of the chronic EEG change, as unmeasured confounds may exist. A single measure of cognitive function was used, while it was a composite measure and thus captured a large domain, the present results do not extend to non-measured domains. For psychological functioning, symptom measures and no diagnoses were used, likely lowering the specificity of effects and emphasizing the tendency to report distress. Finally, the multiple comparisons threshold choice affects outcomes (e.g., effects of PTA and repetitive TBI in gamma band similar to beta but did not meet the threshold for significance after correction).

## 6. Conclusion

The simple history of mTBI does not have long-term effects on resting EEG. However, higher levels of mTBI dose and severity have distinct chronic correlates in higher frequency resting EEG. Cognitive complaints may indicate specific problems with the functioning of this network. Different, slower resting oscillations may underlie difficulties with cognitive processing and psychological distress. In conclusion, the present study illustrates the complexity of even resting-state spectra as a measure of brain injury effects, in the varied influences from remote neurological events to subjective psychological states. Thus, because of the varied effects due to different injury variables and psychological injury correlates, studies of EEG of mTBI must account for more than simple injury status. Beta power reduction in particular may be a useful biomarker of chronic effects of more severe injuries involving PTA and loss of consciousness.

## Data availability statement

The datasets presented in this study can be found in online repositories. The names of the repository/repositories and accession number(s) can be found below: Federal Interagency TBI Repository: https://fitbir.nih.gov/.

## Ethics statement

The studies involving humans were approved by the Virginia Commonwealth University/Richmond VA/Walter Reed National Military Medical Center/Minneapolis VA IRBs. The studies were conducted in accordance with the local legislation and institutional requirements. The participants provided their written informed consent to participate in this study.

## Author contributions

LF: design of study, data acquisition and interpretation, and preparation and critical review of manuscript. RP: statistical design and analysis and preparation and critical review of manuscript. SS: data acquisition and interpretation and critical review of manuscript. All authors contributed to the article and approved the submitted version.
