# Supplementary material for: Long-term resting EEG correlates of repetitive mild traumatic brain injury and loss of consciousness: alterations in alpha-beta power
Source: Front Neurol. 2023 Aug 29;14:1241481. doi: 10.3389/fneur.2023.1241481 (PMC10495577; doi:10.3389/fneur.2023.1241481)
Supplement: Supplementary file 1 [file Data_Sheet_1.docx]

**Supplemental Tables**

**All Regressions of absolute power by Region and EEG band.** Est= b weight estimate, SE= standard error.

1. **Anterior Left Alpha**

|  | | Unadjusted | | | | | | | | Adjusted | | | | | | |  |
| --- | --- | --- | --- | --- | --- | --- | --- | --- | --- | --- | --- | --- | --- | --- | --- | --- | --- |
| Measure | | Est. | | SE | | t | | p | | Est. | | SE | | t | p | |  |
| DRRI2 | | -0.007 | | 0.008 | | -0.917 | | 0.360 | | 0.004 | | 0.011 | | 0.331 | 0.741 | |  |
| TBIQOL Cognition | | 0.011 | | 0.011 | | 0.985 | | 0.326 | | 0.012 | | 0.024 | | 0.521 | 0.603 | |  |
| TBIQOL Executive Function | | 0.003 | | 0.014 | | 0.210 | | 0.834 | | -0.023 | | 0.029 | | -0.777 | 0.438 | |  |
| Fluid Cognition | | -0.005 | | 0.009 | | -0.580 | | 0.562 | | -0.008 | | 0.012 | | -0.703 | 0.483 | |  |
| TOPF | | 0.004 | | 0.010 | | 0.379 | | 0.705 | | 0.008 | | 0.011 | | 0.699 | 0.485 | |  |
| AGE | | 0.007 | | 0.011 | | 0.585 | | 0.559 | | -0.004 | | 0.014 | | -0.305 | 0.761 | |  |
| PCL5 | | 0.001 | | 0.006 | | 0.212 | | 0.832 | | 0.036 | | 0.012 | | 2.909 | 0.004 | |  |
| PHQ9 | | -0.024 | | 0.018 | | -1.278 | | 0.202 | | -0.119 | | 0.040 | | -2.982 | 0.003 | |  |
| TBI Exposure | | NA | | NA | | NA | | NA | | NA | | NA | | NA | NA | |  |
| Unexposed | | NA | | NA | | NA | | NA | | NA | | NA | | NA | NA | |  |
| Combat mTBI | | -0.532 | | 0.282 | | -1.887 | | 0.060 | | -0.904 | | 0.423 | | -2.135 | 0.034 | |  |
| Non-Combat TBI | | -0.189 | | 0.310 | | -0.609 | | 0.543 | | -0.294 | | 0.369 | | -0.796 | 0.427 | |  |
| NUMBER OF BLAST PCES | | -0.077 | | 0.051 | | -1.491 | | 0.137 | | -0.070 | | 0.066 | | -1.048 | 0.296 | |  |
| Number of Positive mTBIs | | -0.049 | | 0.063 | | -0.777 | | 0.438 | | 0.122 | | 0.094 | | 1.295 | 0.196 | |  |
| Number of TBI with PTA+LOC | | -0.290 | | 0.186 | | -1.557 | | 0.120 | | -0.409 | | 0.235 | | -1.738 | 0.083 | |  |
|  |  | |  | |  | |  | |  | |  | |  | | |  | |
|  |  | |  | |  | |  | |  | |  | |  | | |  | |
|  |  | |  | |  | |  | |  | |  | |  | | |  | |

1. **Anterior Left Beta**

|  | Unadjusted | | | | Adjusted | | | |
| --- | --- | --- | --- | --- | --- | --- | --- | --- |
| Measure | Est. | SE | t | p | Est. | SE | t | p |
| DRRI2 | -0.002 | 0.001 | -2.578 | 0.010 | -0.003 | 0.001 | -2.314 | 0.021 |
| TBIQOL Cognition | 0.000 | 0.001 | 0.062 | 0.951 | -0.005 | 0.002 | -2.137 | 0.033 |
| TBIQOL Executive Function | 0.002 | 0.002 | 1.052 | 0.294 | 0.005 | 0.003 | 1.681 | 0.094 |
| Fluid Cognition | 0.001 | 0.001 | 0.637 | 0.524 | 0.001 | 0.001 | 0.606 | 0.545 |
| TOPF | 0.000 | 0.001 | 0.363 | 0.717 | 0.000 | 0.001 | -0.213 | 0.832 |
| AGE | 0.001 | 0.001 | 0.542 | 0.588 | 0.001 | 0.001 | 0.412 | 0.680 |
| PCL5 | 0.000 | 0.001 | -0.264 | 0.792 | 0.002 | 0.001 | 1.585 | 0.114 |
| PHQ9 | -0.003 | 0.002 | -1.353 | 0.177 | -0.007 | 0.004 | -1.593 | 0.112 |
| TBI Exposure | NA | NA | NA | NA | NA | NA | NA | NA |
| Unexposed | NA | NA | NA | NA | NA | NA | NA | NA |
| Combat mTBI | -0.044 | 0.031 | -1.414 | 0.158 | -0.042 | 0.044 | -0.947 | 0.345 |
| Non-Combat TBI | -0.001 | 0.034 | -0.029 | 0.977 | -0.012 | 0.039 | -0.299 | 0.765 |
| NUMBER OF BLAST PCES | -0.004 | 0.006 | -0.777 | 0.438 | 0.002 | 0.007 | 0.352 | 0.725 |
| Number of Positive mTBIs | -0.001 | 0.007 | -0.204 | 0.838 | 0.021 | 0.010 | 2.119 | 0.035 |
| Number of TBI with PTA+LOC | -0.068 | 0.020 | -3.341 | 0.001 | -0.082 | 0.025 | -3.318 | 0.001 |

1. **Anterior Left Delta**

|  | Unadjusted | | | | Adjusted | | | |
| --- | --- | --- | --- | --- | --- | --- | --- | --- |
| Measure | Est. | SE | t | p | Est. | SE | t | p |
| DRRI2 | 0.020 | 0.013 | 1.537 | 0.125 | -0.007 | 0.017 | -0.423 | 0.672 |
| TBIQOL Cognition | -0.037 | 0.018 | -2.024 | 0.044 | 0.010 | 0.035 | 0.271 | 0.787 |
| TBIQOL Executive Function | -0.054 | 0.022 | -2.486 | 0.013 | 0.008 | 0.044 | 0.185 | 0.853 |
| Fluid Cognition | -0.065 | 0.015 | -4.346 | 0.000 | -0.062 | 0.018 | -3.467 | 0.001 |
| TOPF | -0.060 | 0.016 | -3.846 | 0.000 | -0.036 | 0.017 | -2.156 | 0.032 |
| AGE | -0.025 | 0.019 | -1.355 | 0.176 | -0.058 | 0.021 | -2.723 | 0.007 |
| PCL5 | 0.038 | 0.009 | 4.130 | 0.000 | 0.026 | 0.018 | 1.400 | 0.163 |
| PHQ9 | 0.089 | 0.030 | 2.965 | 0.003 | 0.004 | 0.060 | 0.068 | 0.946 |
| TBI Exposure | NA | NA | NA | NA | NA | NA | NA | NA |
| Unexposed | NA | NA | NA | NA | NA | NA | NA | NA |
| Combat mTBI | 0.622 | 0.465 | 1.338 | 0.182 | 0.615 | 0.634 | 0.970 | 0.333 |
| Non-Combat TBI | 0.118 | 0.511 | 0.232 | 0.817 | 0.315 | 0.553 | 0.570 | 0.569 |
| NUMBER OF BLAST PCES | 0.052 | 0.085 | 0.616 | 0.538 | 0.022 | 0.100 | 0.217 | 0.829 |
| Number of Positive mTBIs | 0.074 | 0.104 | 0.717 | 0.474 | 0.004 | 0.141 | 0.031 | 0.976 |
| Number of TBI with PTA+LOC | 0.273 | 0.307 | 0.891 | 0.374 | -0.266 | 0.352 | -0.755 | 0.451 |

1. **Anterior Left Gamma**

|  | Unadjusted | | | | Adjusted | | | |
| --- | --- | --- | --- | --- | --- | --- | --- | --- |
| Measure | Est. | SE | t | p | Est. | SE | t | p |
| DRRI2 | 0.000 | 0.000 | 0.579 | 0.563 | 0.000 | 0.000 | -0.567 | 0.571 |
| TBIQOL Cognition | 0.000 | 0.000 | -1.563 | 0.119 | -0.001 | 0.000 | -1.828 | 0.069 |
| TBIQOL Executive Function | 0.000 | 0.000 | -0.064 | 0.949 | 0.001 | 0.000 | 2.199 | 0.029 |
| Fluid Cognition | 0.000 | 0.000 | -1.219 | 0.224 | 0.000 | 0.000 | -2.179 | 0.030 |
| TOPF | 0.000 | 0.000 | 1.660 | 0.098 | 0.000 | 0.000 | 2.203 | 0.028 |
| AGE | 0.000 | 0.000 | -0.003 | 0.998 | 0.000 | 0.000 | -0.439 | 0.661 |
| PCL5 | 0.000 | 0.000 | -0.172 | 0.864 | 0.000 | 0.000 | -1.038 | 0.300 |
| PHQ9 | 0.000 | 0.000 | 0.283 | 0.777 | 0.000 | 0.001 | 0.959 | 0.338 |
| TBI Exposure | NA | NA | NA | NA | NA | NA | NA | NA |
| Unexposed | NA | NA | NA | NA | NA | NA | NA | NA |
| Combat mTBI | 0.007 | 0.004 | 1.864 | 0.063 | 0.005 | 0.005 | 0.982 | 0.327 |
| Non-Combat TBI | 0.007 | 0.004 | 1.597 | 0.111 | 0.002 | 0.005 | 0.525 | 0.600 |
| NUMBER OF BLAST PCES | 0.002 | 0.001 | 2.154 | 0.032 | 0.001 | 0.001 | 1.282 | 0.201 |
| Number of Positive mTBIs | 0.002 | 0.001 | 2.596 | 0.010 | 0.003 | 0.001 | 2.236 | 0.026 |
| Number of TBI with PTA+LOC | -0.003 | 0.003 | -1.250 | 0.212 | -0.008 | 0.003 | -2.681 | 0.008 |

1. **Anterior Left Theta**

|  | Unadjusted | | | | Adjusted | | | |
| --- | --- | --- | --- | --- | --- | --- | --- | --- |
| Measure | Est. | SE | t | p | Est. | SE | t | p |
| DRRI2 | 0.004 | 0.007 | 0.542 | 0.588 | 0.007 | 0.008 | 0.795 | 0.427 |
| TBIQOL Cognition | -0.008 | 0.009 | -0.989 | 0.323 | 0.006 | 0.018 | 0.353 | 0.724 |
| TBIQOL Executive Function | -0.014 | 0.011 | -1.312 | 0.191 | -0.024 | 0.022 | -1.091 | 0.276 |
| Fluid Cognition | -0.018 | 0.008 | -2.163 | 0.031 | -0.005 | 0.009 | -0.578 | 0.564 |
| TOPF | -0.014 | 0.009 | -1.584 | 0.114 | -0.010 | 0.008 | -1.192 | 0.234 |
| AGE | -0.004 | 0.011 | -0.341 | 0.733 | -0.007 | 0.011 | -0.649 | 0.517 |
| PCL5 | 0.006 | 0.005 | 1.084 | 0.279 | 0.017 | 0.009 | 1.902 | 0.058 |
| PHQ9 | 0.011 | 0.017 | 0.628 | 0.530 | -0.052 | 0.030 | -1.752 | 0.081 |
| TBI Exposure | NA | NA | NA | NA | NA | NA | NA | NA |
| Unexposed | NA | NA | NA | NA | NA | NA | NA | NA |
| Combat mTBI | -0.014 | 0.262 | -0.054 | 0.957 | -0.054 | 0.314 | -0.171 | 0.865 |
| Non-Combat TBI | 0.247 | 0.287 | 0.860 | 0.391 | 0.050 | 0.274 | 0.181 | 0.856 |
| NUMBER OF BLAST PCES | -0.072 | 0.048 | -1.511 | 0.132 | -0.034 | 0.049 | -0.689 | 0.491 |
| Number of Positive mTBIs | -0.046 | 0.058 | -0.791 | 0.429 | -0.044 | 0.070 | -0.629 | 0.530 |
| Number of TBI with PTA+LOC | -0.066 | 0.173 | -0.383 | 0.702 | -0.146 | 0.175 | -0.836 | 0.404 |

|  | Unadjusted | | | | Adjusted | | | |
| --- | --- | --- | --- | --- | --- | --- | --- | --- |
| Measure | Est. | SE | t | p | Est. | SE | t | p |
| DRRI2 | -0.007 | 0.010 | -0.661 | 0.509 | 0.004 | 0.014 | 0.303 | 0.762 |
| TBIQOL Cognition | 0.012 | 0.014 | 0.850 | 0.396 | 0.013 | 0.030 | 0.424 | 0.672 |
| TBIQOL Executive Function | 0.004 | 0.018 | 0.196 | 0.844 | -0.022 | 0.038 | -0.586 | 0.558 |
| Fluid Cognition | -0.008 | 0.012 | -0.693 | 0.489 | -0.013 | 0.015 | -0.848 | 0.397 |
| TOPF | 0.006 | 0.012 | 0.488 | 0.626 | 0.012 | 0.014 | 0.858 | 0.392 |
| AGE | 0.007 | 0.015 | 0.502 | 0.616 | -0.007 | 0.018 | -0.386 | 0.700 |
| PCL5 | 0.003 | 0.007 | 0.418 | 0.676 | 0.049 | 0.016 | 3.066 | 0.002 |
| PHQ9 | -0.028 | 0.024 | -1.175 | 0.241 | -0.154 | 0.051 | -3.005 | 0.003 |
| TBI Exposure | NA | NA | NA | NA | NA | NA | NA | NA |
| Unexposed | NA | NA | NA | NA | NA | NA | NA | NA |
| Combat mTBI | -0.616 | 0.362 | -1.699 | 0.090 | -1.108 | 0.543 | -2.038 | 0.042 |
| Non-Combat TBI | -0.290 | 0.398 | -0.730 | 0.466 | -0.435 | 0.474 | -0.917 | 0.360 |
| NUMBER OF BLAST PCES | -0.082 | 0.066 | -1.235 | 0.218 | -0.074 | 0.085 | -0.866 | 0.387 |
| Number of Positive mTBIs | -0.057 | 0.081 | -0.701 | 0.484 | 0.150 | 0.121 | 1.241 | 0.216 |
| Number of TBI with PTA+LOC | -0.363 | 0.239 | -1.519 | 0.130 | -0.539 | 0.302 | -1.786 | 0.075 |

1. **Anterior Midline Alpha**
2. **Anterior Midline Beta**

|  | Unadjusted | | | | Adjusted | | | |
| --- | --- | --- | --- | --- | --- | --- | --- | --- |
| Measure | Est. | SE | t | p | Est. | SE | t | p |
| DRRI2 | -0.002 | 0.001 | -1.810 | 0.071 | -0.003 | 0.001 | -1.986 | 0.048 |
| TBIQOL Cognition | 0.000 | 0.001 | 0.082 | 0.934 | -0.005 | 0.003 | -1.889 | 0.060 |
| TBIQOL Executive Function | 0.002 | 0.002 | 1.008 | 0.314 | 0.005 | 0.003 | 1.430 | 0.154 |
| Fluid Cognition | 0.000 | 0.001 | -0.279 | 0.780 | 0.000 | 0.001 | -0.204 | 0.839 |
| TOPF | 0.000 | 0.001 | -0.053 | 0.958 | -0.001 | 0.001 | -0.377 | 0.707 |
| AGE | 0.001 | 0.001 | 0.409 | 0.683 | 0.000 | 0.002 | -0.104 | 0.917 |
| PCL5 | 0.000 | 0.001 | 0.474 | 0.635 | 0.003 | 0.001 | 2.102 | 0.036 |
| PHQ9 | -0.002 | 0.002 | -1.082 | 0.280 | -0.009 | 0.005 | -1.971 | 0.050 |
| TBI Exposure | NA | NA | NA | NA | NA | NA | NA | NA |
| Unexposed | NA | NA | NA | NA | NA | NA | NA | NA |
| Combat mTBI | -0.046 | 0.036 | -1.275 | 0.203 | -0.052 | 0.050 | -1.041 | 0.299 |
| Non-Combat TBI | -0.010 | 0.040 | -0.241 | 0.810 | -0.004 | 0.044 | -0.089 | 0.929 |
| NUMBER OF BLAST PCES | -0.004 | 0.007 | -0.602 | 0.548 | 0.003 | 0.008 | 0.402 | 0.688 |
| Number of Positive mTBIs | -0.005 | 0.008 | -0.606 | 0.545 | 0.015 | 0.011 | 1.345 | 0.180 |
| Number of TBI with PTA+LOC | -0.072 | 0.024 | -3.058 | 0.002 | -0.079 | 0.028 | -2.828 | 0.005 |

1. **Anterior Midline Delta**

|  | Unadjusted | | | | Adjusted | | | |
| --- | --- | --- | --- | --- | --- | --- | --- | --- |
| Measure | Est. | SE | t | p | Est. | SE | t | p |
| DRRI2 | 0.027 | 0.016 | 1.757 | 0.080 | -0.006 | 0.021 | -0.299 | 0.766 |
| TBIQOL Cognition | -0.049 | 0.022 | -2.206 | 0.028 | 0.015 | 0.043 | 0.352 | 0.725 |
| TBIQOL Executive Function | -0.076 | 0.027 | -2.824 | 0.005 | -0.002 | 0.054 | -0.035 | 0.972 |
| Fluid Cognition | -0.083 | 0.018 | -4.575 | 0.000 | -0.064 | 0.022 | -2.900 | 0.004 |
| TOPF | -0.083 | 0.019 | -4.410 | 0.000 | -0.052 | 0.021 | -2.519 | 0.012 |
| AGE | -0.018 | 0.023 | -0.781 | 0.436 | -0.055 | 0.026 | -2.124 | 0.035 |
| PCL5 | 0.058 | 0.011 | 5.159 | 0.000 | 0.054 | 0.023 | 2.375 | 0.018 |
| PHQ9 | 0.125 | 0.036 | 3.460 | 0.001 | -0.043 | 0.073 | -0.588 | 0.557 |
| TBI Exposure | NA | NA | NA | NA | NA | NA | NA | NA |
| Unexposed | NA | NA | NA | NA | NA | NA | NA | NA |
| Combat mTBI | 0.629 | 0.564 | 1.115 | 0.266 | 0.187 | 0.778 | 0.241 | 0.810 |
| Non-Combat TBI | -0.163 | 0.620 | -0.262 | 0.793 | 0.064 | 0.678 | 0.094 | 0.925 |
| NUMBER OF BLAST PCES | 0.074 | 0.103 | 0.714 | 0.475 | 0.071 | 0.122 | 0.585 | 0.559 |
| Number of Positive mTBIs | 0.086 | 0.126 | 0.687 | 0.493 | -0.075 | 0.173 | -0.433 | 0.665 |
| Number of TBI with PTA+LOC | 0.807 | 0.370 | 2.178 | 0.030 | 0.311 | 0.432 | 0.719 | 0.473 |

1. **Anterior Midline Gamma**

|  | Unadjusted | | | | Adjusted | | | |
| --- | --- | --- | --- | --- | --- | --- | --- | --- |
| Measure | Est. | SE | t | p | Est. | SE | t | p |
| DRRI2 | 0.000 | 0.000 | 1.548 | 0.123 | 0.000 | 0.000 | 0.687 | 0.493 |
| TBIQOL Cognition | 0.000 | 0.000 | -1.148 | 0.252 | 0.000 | 0.000 | -2.004 | 0.046 |
| TBIQOL Executive Function | 0.000 | 0.000 | 0.559 | 0.577 | 0.000 | 0.000 | 1.552 | 0.122 |
| Fluid Cognition | 0.000 | 0.000 | 0.366 | 0.715 | 0.000 | 0.000 | -0.709 | 0.479 |
| TOPF | 0.000 | 0.000 | 2.731 | 0.007 | 0.000 | 0.000 | 2.342 | 0.020 |
| AGE | 0.000 | 0.000 | -0.519 | 0.604 | 0.000 | 0.000 | -0.121 | 0.904 |
| PCL5 | 0.000 | 0.000 | -1.367 | 0.173 | 0.000 | 0.000 | -1.164 | 0.245 |
| PHQ9 | 0.000 | 0.000 | -0.941 | 0.347 | 0.000 | 0.000 | -0.017 | 0.986 |
| TBI Exposure | NA | NA | NA | NA | NA | NA | NA | NA |
| Unexposed | NA | NA | NA | NA | NA | NA | NA | NA |
| Combat mTBI | 0.005 | 0.003 | 1.816 | 0.070 | 0.004 | 0.004 | 1.081 | 0.281 |
| Non-Combat TBI | 0.002 | 0.003 | 0.784 | 0.434 | 0.001 | 0.003 | 0.324 | 0.746 |
| NUMBER OF BLAST PCES | 0.001 | 0.000 | 1.711 | 0.088 | 0.000 | 0.001 | 0.408 | 0.684 |
| Number of Positive mTBIs | 0.001 | 0.001 | 2.032 | 0.043 | 0.001 | 0.001 | 1.223 | 0.222 |
| Number of TBI with PTA+LOC | -0.002 | 0.002 | -1.331 | 0.184 | -0.005 | 0.002 | -2.055 | 0.041 |

|  | Unadjusted | | | | Adjusted | | | |
| --- | --- | --- | --- | --- | --- | --- | --- | --- |
| Measure | Est. | SE | t | p | Est. | SE | t | p |
| DRRI2 | 0.012 | 0.009 | 1.346 | 0.179 | 0.010 | 0.011 | 0.859 | 0.391 |
| TBIQOL Cognition | -0.018 | 0.012 | -1.515 | 0.131 | 0.012 | 0.024 | 0.521 | 0.603 |
| TBIQOL Executive Function | -0.028 | 0.015 | -1.898 | 0.059 | -0.037 | 0.030 | -1.262 | 0.208 |
| Fluid Cognition | -0.027 | 0.010 | -2.600 | 0.010 | -0.013 | 0.012 | -1.063 | 0.288 |
| TOPF | -0.020 | 0.011 | -1.794 | 0.074 | -0.014 | 0.011 | -1.195 | 0.233 |
| AGE | -0.010 | 0.013 | -0.725 | 0.469 | -0.016 | 0.014 | -1.121 | 0.263 |
| PCL5 | 0.014 | 0.007 | 2.021 | 0.044 | 0.027 | 0.012 | 2.160 | 0.032 |
| PHQ9 | 0.027 | 0.021 | 1.239 | 0.216 | -0.073 | 0.040 | -1.795 | 0.074 |
| TBI Exposure | NA | NA | NA | NA | NA | NA | NA | NA |
| Unexposed | NA | NA | NA | NA | NA | NA | NA | NA |
| Combat mTBI | 0.138 | 0.330 | 0.418 | 0.677 | -0.032 | 0.428 | -0.075 | 0.941 |
| Non-Combat TBI | 0.283 | 0.363 | 0.782 | 0.435 | 0.083 | 0.373 | 0.224 | 0.823 |
| NUMBER OF BLAST PCES | -0.054 | 0.060 | -0.901 | 0.368 | -0.019 | 0.067 | -0.276 | 0.783 |
| Number of Positive mTBIs | -0.029 | 0.073 | -0.395 | 0.693 | -0.071 | 0.095 | -0.746 | 0.456 |
| Number of TBI with PTA+LOC | 0.052 | 0.218 | 0.238 | 0.812 | -0.122 | 0.238 | -0.513 | 0.608 |

1. **Anterior Midline Theta**
2. **Anterior Right Alpha**

|  | Unadjusted | | | | Adjusted | | | |
| --- | --- | --- | --- | --- | --- | --- | --- | --- |
| Measure | Est. | SE | t | p | Est. | SE | t | p |
| DRRI2 | -0.008 | 0.008 | -0.952 | 0.342 | 0.003 | 0.012 | 0.268 | 0.789 |
| TBIQOL Cognition | 0.012 | 0.012 | 1.034 | 0.302 | 0.009 | 0.025 | 0.371 | 0.711 |
| TBIQOL Executive Function | 0.004 | 0.015 | 0.307 | 0.759 | -0.018 | 0.031 | -0.593 | 0.553 |
| Fluid Cognition | -0.007 | 0.010 | -0.668 | 0.505 | -0.011 | 0.012 | -0.901 | 0.369 |
| TOPF | 0.005 | 0.010 | 0.511 | 0.610 | 0.010 | 0.012 | 0.892 | 0.373 |
| AGE | 0.005 | 0.012 | 0.449 | 0.654 | -0.008 | 0.015 | -0.506 | 0.613 |
| PCL5 | 0.001 | 0.006 | 0.133 | 0.894 | 0.036 | 0.013 | 2.770 | 0.006 |
| PHQ9 | -0.026 | 0.019 | -1.334 | 0.183 | -0.123 | 0.042 | -2.930 | 0.004 |
| TBI Exposure | NA | NA | NA | NA | NA | NA | NA | NA |
| Unexposed | NA | NA | NA | NA | NA | NA | NA | NA |
| Combat mTBI | -0.554 | 0.294 | -1.882 | 0.061 | -0.995 | 0.443 | -2.247 | 0.025 |
| Non-Combat TBI | -0.273 | 0.323 | -0.843 | 0.400 | -0.420 | 0.386 | -1.087 | 0.278 |
| NUMBER OF BLAST PCES | -0.079 | 0.054 | -1.475 | 0.141 | -0.076 | 0.069 | -1.097 | 0.273 |
| Number of Positive mTBIs | -0.041 | 0.066 | -0.631 | 0.529 | 0.137 | 0.098 | 1.391 | 0.165 |
| Number of TBI with PTA+LOC | -0.238 | 0.195 | -1.223 | 0.222 | -0.347 | 0.246 | -1.409 | 0.160 |

1. **Anterior Right Beta**

|  | Unadjusted | | | | Adjusted | | | |
| --- | --- | --- | --- | --- | --- | --- | --- | --- |
| Measure | Est. | SE | t | p | Est. | SE | t | p |
| DRRI2 | -0.002 | 0.001 | -2.291 | 0.023 | -0.003 | 0.001 | -2.337 | 0.020 |
| TBIQOL Cognition | 0.000 | 0.001 | -0.139 | 0.889 | -0.005 | 0.003 | -1.914 | 0.057 |
| TBIQOL Executive Function | 0.001 | 0.002 | 0.597 | 0.551 | 0.004 | 0.003 | 1.383 | 0.168 |
| Fluid Cognition | 0.000 | 0.001 | -0.091 | 0.928 | 0.000 | 0.001 | -0.042 | 0.967 |
| TOPF | 0.000 | 0.001 | 0.312 | 0.755 | 0.000 | 0.001 | 0.170 | 0.865 |
| AGE | 0.000 | 0.001 | 0.006 | 0.995 | 0.000 | 0.002 | -0.311 | 0.756 |
| PCL5 | 0.000 | 0.001 | 0.605 | 0.546 | 0.002 | 0.001 | 1.719 | 0.087 |
| PHQ9 | -0.002 | 0.002 | -0.835 | 0.405 | -0.007 | 0.004 | -1.614 | 0.108 |
| TBI Exposure | NA | NA | NA | NA | NA | NA | NA | NA |
| Unexposed | NA | NA | NA | NA | NA | NA | NA | NA |
| Combat mTBI | -0.038 | 0.033 | -1.149 | 0.251 | -0.050 | 0.045 | -1.100 | 0.272 |
| Non-Combat TBI | -0.016 | 0.037 | -0.430 | 0.668 | -0.023 | 0.040 | -0.591 | 0.555 |
| NUMBER OF BLAST PCES | -0.006 | 0.006 | -1.040 | 0.299 | -0.001 | 0.007 | -0.119 | 0.906 |
| Number of Positive mTBIs | 0.000 | 0.007 | -0.040 | 0.968 | 0.021 | 0.010 | 2.049 | 0.041 |
| Number of TBI with PTA+LOC | -0.052 | 0.022 | -2.393 | 0.017 | -0.058 | 0.025 | -2.310 | 0.022 |

1. **Anterior Right Delta**

|  | Unadjusted | | | | Adjusted | | | |
| --- | --- | --- | --- | --- | --- | --- | --- | --- |
| Measure | Est. | SE | t | p | Est. | SE | t | p |
| DRRI2 | 0.018 | 0.013 | 1.391 | 0.165 | -0.008 | 0.018 | -0.447 | 0.655 |
| TBIQOL Cognition | -0.037 | 0.019 | -1.932 | 0.054 | 0.010 | 0.038 | 0.259 | 0.796 |
| TBIQOL Executive Function | -0.054 | 0.023 | -2.369 | 0.018 | 0.006 | 0.047 | 0.132 | 0.895 |
| Fluid Cognition | -0.071 | 0.015 | -4.591 | 0.000 | -0.069 | 0.019 | -3.666 | 0.000 |
| TOPF | -0.059 | 0.016 | -3.669 | 0.000 | -0.034 | 0.018 | -1.919 | 0.056 |
| AGE | -0.024 | 0.019 | -1.223 | 0.222 | -0.061 | 0.023 | -2.684 | 0.008 |
| PCL5 | 0.039 | 0.010 | 4.093 | 0.000 | 0.025 | 0.020 | 1.265 | 0.207 |
| PHQ9 | 0.091 | 0.031 | 2.937 | 0.004 | 0.001 | 0.063 | 0.013 | 0.990 |
| TBI Exposure | NA | NA | NA | NA | NA | NA | NA | NA |
| Unexposed | NA | NA | NA | NA | NA | NA | NA | NA |
| Combat mTBI | 0.540 | 0.481 | 1.124 | 0.262 | 0.457 | 0.672 | 0.681 | 0.497 |
| Non-Combat TBI | 0.100 | 0.528 | 0.190 | 0.850 | 0.281 | 0.586 | 0.479 | 0.632 |
| NUMBER OF BLAST PCES | 0.043 | 0.088 | 0.495 | 0.621 | 0.033 | 0.105 | 0.308 | 0.758 |
| Number of Positive mTBIs | 0.059 | 0.107 | 0.548 | 0.584 | -0.033 | 0.149 | -0.221 | 0.825 |
| Number of TBI with PTA+LOC | 0.354 | 0.317 | 1.117 | 0.265 | -0.125 | 0.373 | -0.335 | 0.738 |

1. **Anterior Right Gamma**

|  | Unadjusted | | | | Adjusted | | | |
| --- | --- | --- | --- | --- | --- | --- | --- | --- |
| Measure | Est. | SE | t | p | Est. | SE | t | p |
| DRRI2 | 0.000 | 0.000 | 0.787 | 0.432 | 0.000 | 0.000 | 0.151 | 0.880 |
| TBIQOL Cognition | 0.000 | 0.000 | -1.100 | 0.272 | 0.000 | 0.000 | -1.549 | 0.123 |
| TBIQOL Executive Function | 0.000 | 0.000 | 0.132 | 0.895 | 0.001 | 0.000 | 1.553 | 0.121 |
| Fluid Cognition | 0.000 | 0.000 | -0.482 | 0.630 | 0.000 | 0.000 | -1.033 | 0.303 |
| TOPF | 0.000 | 0.000 | 2.380 | 0.018 | 0.000 | 0.000 | 2.380 | 0.018 |
| AGE | 0.000 | 0.000 | 0.204 | 0.839 | 0.000 | 0.000 | 0.295 | 0.769 |
| PCL5 | 0.000 | 0.000 | -0.536 | 0.592 | 0.000 | 0.000 | -0.577 | 0.564 |
| PHQ9 | 0.000 | 0.000 | -0.574 | 0.566 | 0.000 | 0.001 | 0.010 | 0.992 |
| TBI Exposure | NA | NA | NA | NA | NA | NA | NA | NA |
| Unexposed | NA | NA | NA | NA | NA | NA | NA | NA |
| Combat mTBI | 0.005 | 0.004 | 1.393 | 0.165 | 0.002 | 0.005 | 0.309 | 0.757 |
| Non-Combat TBI | 0.003 | 0.004 | 0.812 | 0.417 | 0.000 | 0.005 | -0.042 | 0.966 |
| NUMBER OF BLAST PCES | 0.001 | 0.001 | 1.588 | 0.113 | 0.000 | 0.001 | 0.327 | 0.744 |
| Number of Positive mTBIs | 0.002 | 0.001 | 2.819 | 0.005 | 0.003 | 0.001 | 2.764 | 0.006 |
| Number of TBI with PTA+LOC | -0.003 | 0.002 | -1.040 | 0.299 | -0.006 | 0.003 | -2.068 | 0.040 |

1. **Anterior Right Theta**

|  | Unadjusted | | | | Adjusted | | | |
| --- | --- | --- | --- | --- | --- | --- | --- | --- |
| Measure | Est. | SE | t | p | Est. | SE | t | p |
| DRRI2 | 0.004 | 0.007 | 0.657 | 0.512 | 0.005 | 0.008 | 0.645 | 0.519 |
| TBIQOL Cognition | -0.009 | 0.008 | -1.072 | 0.284 | 0.007 | 0.017 | 0.403 | 0.687 |
| TBIQOL Executive Function | -0.017 | 0.011 | -1.589 | 0.113 | -0.028 | 0.022 | -1.305 | 0.193 |
| Fluid Cognition | -0.019 | 0.008 | -2.426 | 0.016 | -0.009 | 0.009 | -1.025 | 0.306 |
| TOPF | -0.013 | 0.008 | -1.592 | 0.112 | -0.009 | 0.008 | -1.057 | 0.292 |
| AGE | -0.006 | 0.010 | -0.589 | 0.556 | -0.011 | 0.010 | -1.066 | 0.287 |
| PCL5 | 0.007 | 0.005 | 1.513 | 0.131 | 0.017 | 0.009 | 1.873 | 0.062 |
| PHQ9 | 0.012 | 0.016 | 0.786 | 0.432 | -0.056 | 0.029 | -1.892 | 0.060 |
| TBI Exposure | NA | NA | NA | NA | NA | NA | NA | NA |
| Unexposed | NA | NA | NA | NA | NA | NA | NA | NA |
| Combat mTBI | 0.031 | 0.239 | 0.132 | 0.895 | -0.019 | 0.311 | -0.062 | 0.951 |
| Non-Combat TBI | 0.165 | 0.262 | 0.630 | 0.529 | 0.036 | 0.271 | 0.134 | 0.893 |
| NUMBER OF BLAST PCES | -0.064 | 0.043 | -1.472 | 0.142 | -0.032 | 0.049 | -0.645 | 0.519 |
| Number of Positive mTBIs | -0.035 | 0.053 | -0.661 | 0.509 | -0.058 | 0.069 | -0.841 | 0.401 |
| Number of TBI with PTA+LOC | 0.010 | 0.157 | 0.062 | 0.951 | -0.067 | 0.173 | -0.390 | 0.697 |

1. **Central Left Alpha**

|  | Unadjusted | | | | Adjusted | | | |
| --- | --- | --- | --- | --- | --- | --- | --- | --- |
| Measure | Est. | SE | t | p | Est. | SE | t | p |
| DRRI2 | -0.006 | 0.008 | -0.791 | 0.430 | 0.005 | 0.011 | 0.401 | 0.688 |
| TBIQOL Cognition | 0.008 | 0.011 | 0.704 | 0.482 | 0.011 | 0.024 | 0.459 | 0.646 |
| TBIQOL Executive Function | -0.002 | 0.014 | -0.157 | 0.875 | -0.030 | 0.030 | -1.007 | 0.315 |
| Fluid Cognition | -0.002 | 0.010 | -0.257 | 0.798 | -0.002 | 0.012 | -0.129 | 0.898 |
| TOPF | 0.006 | 0.010 | 0.654 | 0.514 | 0.010 | 0.011 | 0.852 | 0.395 |
| AGE | 0.004 | 0.012 | 0.354 | 0.723 | -0.004 | 0.014 | -0.260 | 0.795 |
| PCL5 | 0.002 | 0.006 | 0.282 | 0.778 | 0.037 | 0.012 | 2.953 | 0.003 |
| PHQ9 | -0.020 | 0.019 | -1.073 | 0.284 | -0.127 | 0.040 | -3.151 | 0.002 |
| TBI Exposure | NA | NA | NA | NA | NA | NA | NA | NA |
| Unexposed | NA | NA | NA | NA | NA | NA | NA | NA |
| Combat mTBI | -0.401 | 0.289 | -1.387 | 0.166 | -0.681 | 0.426 | -1.600 | 0.111 |
| Non-Combat TBI | 0.047 | 0.317 | 0.148 | 0.883 | -0.024 | 0.371 | -0.064 | 0.949 |
| NUMBER OF BLAST PCES | -0.095 | 0.053 | -1.803 | 0.072 | -0.085 | 0.067 | -1.269 | 0.206 |
| Number of Positive mTBIs | -0.038 | 0.065 | -0.588 | 0.557 | 0.105 | 0.094 | 1.109 | 0.269 |
| Number of TBI with PTA+LOC | -0.261 | 0.191 | -1.369 | 0.172 | -0.490 | 0.237 | -2.073 | 0.039 |

|  | Unadjusted | | | | Adjusted | | | |
| --- | --- | --- | --- | --- | --- | --- | --- | --- |
| Measure | Est. | SE | t | p | Est. | SE | t | p |
| DRRI2 | -0.002 | 0.001 | -2.541 | 0.012 | -0.003 | 0.001 | -2.319 | 0.021 |
| TBIQOL Cognition | 0.000 | 0.001 | -0.253 | 0.800 | -0.006 | 0.003 | -2.504 | 0.013 |
| TBIQOL Executive Function | 0.001 | 0.002 | 0.847 | 0.398 | 0.005 | 0.003 | 1.600 | 0.111 |
| Fluid Cognition | 0.002 | 0.001 | 1.919 | 0.056 | 0.003 | 0.001 | 2.334 | 0.020 |
| TOPF | 0.000 | 0.001 | 0.398 | 0.691 | -0.001 | 0.001 | -1.097 | 0.274 |
| AGE | 0.002 | 0.001 | 1.188 | 0.236 | 0.003 | 0.002 | 1.708 | 0.089 |
| PCL5 | -0.001 | 0.001 | -1.032 | 0.303 | 0.000 | 0.001 | 0.250 | 0.803 |
| PHQ9 | -0.003 | 0.002 | -1.387 | 0.166 | -0.003 | 0.004 | -0.672 | 0.502 |
| TBI Exposure | NA | NA | NA | NA | NA | NA | NA | NA |
| Unexposed | NA | NA | NA | NA | NA | NA | NA | NA |
| Combat mTBI | -0.037 | 0.033 | -1.126 | 0.261 | -0.013 | 0.046 | -0.284 | 0.777 |
| Non-Combat TBI | -0.006 | 0.036 | -0.166 | 0.868 | -0.021 | 0.040 | -0.528 | 0.598 |
| NUMBER OF BLAST PCES | -0.006 | 0.006 | -1.079 | 0.282 | -0.002 | 0.007 | -0.266 | 0.790 |
| Number of Positive mTBIs | 0.001 | 0.007 | 0.161 | 0.873 | 0.025 | 0.010 | 2.463 | 0.014 |
| Number of TBI with PTA+LOC | -0.085 | 0.021 | -4.042 | 0.000 | -0.107 | 0.025 | -4.231 | 0.000 |

1. **Central Left Beta**
2. **Central Left Delta**

|  | Unadjusted | | | | Adjusted | | | |
| --- | --- | --- | --- | --- | --- | --- | --- | --- |
| Measure | Est. | SE | t | p | Est. | SE | t | p |
| DRRI2 | 0.016 | 0.008 | 1.949 | 0.052 | 0.004 | 0.011 | 0.328 | 0.743 |
| TBIQOL Cognition | -0.025 | 0.012 | -2.059 | 0.040 | 0.009 | 0.023 | 0.370 | 0.711 |
| TBIQOL Executive Function | -0.037 | 0.014 | -2.606 | 0.010 | -0.003 | 0.029 | -0.105 | 0.917 |
| Fluid Cognition | -0.035 | 0.010 | -3.630 | 0.000 | -0.020 | 0.012 | -1.740 | 0.083 |
| TOPF | -0.042 | 0.010 | -4.133 | 0.000 | -0.026 | 0.011 | -2.355 | 0.019 |
| AGE | -0.012 | 0.012 | -0.972 | 0.332 | -0.022 | 0.014 | -1.587 | 0.114 |
| PCL5 | 0.031 | 0.006 | 5.189 | 0.000 | 0.040 | 0.012 | 3.294 | 0.001 |
| PHQ9 | 0.058 | 0.019 | 3.009 | 0.003 | -0.053 | 0.039 | -1.359 | 0.175 |
| TBI Exposure | NA | NA | NA | NA | NA | NA | NA | NA |
| Unexposed | NA | NA | NA | NA | NA | NA | NA | NA |
| Combat mTBI | 0.300 | 0.301 | 0.996 | 0.320 | 0.087 | 0.415 | 0.209 | 0.834 |
| Non-Combat TBI | -0.154 | 0.331 | -0.465 | 0.643 | -0.056 | 0.362 | -0.155 | 0.877 |
| NUMBER OF BLAST PCES | -0.023 | 0.055 | -0.423 | 0.673 | -0.046 | 0.065 | -0.707 | 0.480 |
| Number of Positive mTBIs | 0.035 | 0.067 | 0.515 | 0.607 | 0.000 | 0.092 | 0.001 | 0.999 |
| Number of TBI with PTA+LOC | 0.283 | 0.198 | 1.428 | 0.154 | -0.022 | 0.230 | -0.095 | 0.925 |

1. **Central Left Gamma**

|  | Unadjusted | | | | Adjusted | | | |
| --- | --- | --- | --- | --- | --- | --- | --- | --- |
| Measure | Est. | SE | t | p | Est. | SE | t | p |
| DRRI2 | 0.000 | 0.000 | -0.089 | 0.929 | 0.000 | 0.000 | -1.859 | 0.064 |
| TBIQOL Cognition | 0.000 | 0.000 | -1.931 | 0.054 | -0.001 | 0.000 | -1.689 | 0.092 |
| TBIQOL Executive Function | 0.000 | 0.000 | -0.525 | 0.600 | 0.001 | 0.001 | 2.201 | 0.029 |
| Fluid Cognition | 0.000 | 0.000 | -0.862 | 0.389 | 0.000 | 0.000 | -0.613 | 0.540 |
| TOPF | 0.000 | 0.000 | 0.724 | 0.469 | 0.000 | 0.000 | 0.778 | 0.437 |
| AGE | 0.000 | 0.000 | 1.284 | 0.200 | 0.000 | 0.000 | 1.134 | 0.258 |
| PCL5 | 0.000 | 0.000 | 0.430 | 0.667 | 0.000 | 0.000 | -1.228 | 0.220 |
| PHQ9 | 0.000 | 0.000 | 1.311 | 0.191 | 0.001 | 0.001 | 1.972 | 0.050 |
| TBI Exposure | NA | NA | NA | NA | NA | NA | NA | NA |
| Unexposed | NA | NA | NA | NA | NA | NA | NA | NA |
| Combat mTBI | 0.011 | 0.005 | 2.221 | 0.027 | 0.011 | 0.007 | 1.497 | 0.136 |
| Non-Combat TBI | 0.006 | 0.006 | 1.002 | 0.317 | -0.001 | 0.007 | -0.130 | 0.897 |
| NUMBER OF BLAST PCES | 0.002 | 0.001 | 1.963 | 0.051 | 0.002 | 0.001 | 1.474 | 0.142 |
| Number of Positive mTBIs | 0.003 | 0.001 | 3.000 | 0.003 | 0.004 | 0.002 | 2.482 | 0.014 |
| Number of TBI with PTA+LOC | -0.006 | 0.003 | -1.819 | 0.070 | -0.014 | 0.004 | -3.371 | 0.001 |

1. **Central Left Theta**

|  | Unadjusted | | | | Adjusted | | | |
| --- | --- | --- | --- | --- | --- | --- | --- | --- |
| Measure | Est. | SE | t | p | Est. | SE | t | p |
| DRRI2 | 0.009 | 0.007 | 1.326 | 0.186 | 0.012 | 0.008 | 1.432 | 0.153 |
| TBIQOL Cognition | -0.011 | 0.009 | -1.183 | 0.238 | -0.001 | 0.018 | -0.065 | 0.948 |
| TBIQOL Executive Function | -0.012 | 0.011 | -1.101 | 0.272 | -0.017 | 0.022 | -0.777 | 0.438 |
| Fluid Cognition | -0.012 | 0.008 | -1.553 | 0.122 | 0.004 | 0.009 | 0.464 | 0.643 |
| TOPF | -0.013 | 0.008 | -1.559 | 0.120 | -0.012 | 0.008 | -1.487 | 0.138 |
| AGE | -0.001 | 0.010 | -0.091 | 0.928 | 0.004 | 0.011 | 0.341 | 0.733 |
| PCL5 | 0.007 | 0.005 | 1.379 | 0.169 | 0.020 | 0.009 | 2.181 | 0.030 |
| PHQ9 | 0.008 | 0.016 | 0.521 | 0.603 | -0.060 | 0.030 | -2.006 | 0.046 |
| TBI Exposure | NA | NA | NA | NA | NA | NA | NA | NA |
| Unexposed | NA | NA | NA | NA | NA | NA | NA | NA |
| Combat mTBI | 0.005 | 0.248 | 0.020 | 0.984 | -0.022 | 0.317 | -0.070 | 0.944 |
| Non-Combat TBI | 0.204 | 0.273 | 0.747 | 0.456 | 0.060 | 0.276 | 0.216 | 0.829 |
| NUMBER OF BLAST PCES | -0.089 | 0.045 | -1.976 | 0.049 | -0.076 | 0.050 | -1.520 | 0.130 |
| Number of Positive mTBIs | -0.053 | 0.055 | -0.955 | 0.340 | -0.044 | 0.070 | -0.631 | 0.529 |
| Number of TBI with PTA+LOC | -0.048 | 0.164 | -0.294 | 0.769 | -0.161 | 0.176 | -0.914 | 0.362 |

1. **Central Midline Alpha**

|  | Unadjusted | | | | Adjusted | | | |
| --- | --- | --- | --- | --- | --- | --- | --- | --- |
| Measure | Est. | SE | t | p | Est. | SE | t | p |
| DRRI2 | -0.008 | 0.010 | -0.753 | 0.452 | 0.001 | 0.015 | 0.093 | 0.926 |
| TBIQOL Cognition | 0.009 | 0.015 | 0.627 | 0.531 | 0.011 | 0.031 | 0.367 | 0.714 |
| TBIQOL Executive Function | -0.001 | 0.019 | -0.040 | 0.968 | -0.031 | 0.039 | -0.805 | 0.421 |
| Fluid Cognition | -0.004 | 0.013 | -0.336 | 0.737 | -0.007 | 0.016 | -0.464 | 0.643 |
| TOPF | 0.010 | 0.013 | 0.782 | 0.435 | 0.014 | 0.015 | 0.978 | 0.329 |
| AGE | 0.002 | 0.015 | 0.161 | 0.872 | -0.012 | 0.019 | -0.614 | 0.540 |
| PCL5 | 0.003 | 0.008 | 0.341 | 0.734 | 0.053 | 0.016 | 3.256 | 0.001 |
| PHQ9 | -0.029 | 0.025 | -1.175 | 0.241 | -0.177 | 0.053 | -3.351 | 0.001 |
| TBI Exposure | NA | NA | NA | NA | NA | NA | NA | NA |
| Unexposed | NA | NA | NA | NA | NA | NA | NA | NA |
| Combat mTBI | -0.499 | 0.377 | -1.322 | 0.187 | -0.932 | 0.558 | -1.670 | 0.096 |
| Non-Combat TBI | 0.045 | 0.415 | 0.109 | 0.913 | -0.093 | 0.487 | -0.191 | 0.849 |
| NUMBER OF BLAST PCES | -0.095 | 0.069 | -1.376 | 0.170 | -0.073 | 0.088 | -0.829 | 0.408 |
| Number of Positive mTBIs | -0.037 | 0.084 | -0.435 | 0.664 | 0.161 | 0.124 | 1.299 | 0.195 |
| Number of TBI with PTA+LOC | -0.386 | 0.249 | -1.552 | 0.122 | -0.699 | 0.310 | -2.254 | 0.025 |

1. **Central Midline Beta**

|  | Unadjusted | | | | Adjusted | | | |
| --- | --- | --- | --- | --- | --- | --- | --- | --- |
| Measure | Est. | SE | t | p | Est. | SE | t | p |
| DRRI2 | -0.002 | 0.001 | -1.713 | 0.088 | -0.002 | 0.001 | -1.903 | 0.058 |
| TBIQOL Cognition | 0.000 | 0.001 | 0.059 | 0.953 | -0.006 | 0.003 | -2.102 | 0.036 |
| TBIQOL Executive Function | 0.001 | 0.002 | 0.912 | 0.362 | 0.003 | 0.003 | 1.038 | 0.300 |
| Fluid Cognition | 0.002 | 0.001 | 1.650 | 0.100 | 0.003 | 0.001 | 2.019 | 0.044 |
| TOPF | 0.000 | 0.001 | 0.193 | 0.847 | -0.001 | 0.001 | -1.144 | 0.254 |
| AGE | 0.001 | 0.001 | 0.629 | 0.530 | 0.002 | 0.002 | 0.934 | 0.351 |
| PCL5 | 0.000 | 0.001 | -0.130 | 0.897 | 0.002 | 0.001 | 1.718 | 0.087 |
| PHQ9 | -0.004 | 0.002 | -1.575 | 0.116 | -0.009 | 0.005 | -2.052 | 0.041 |
| TBI Exposure | NA | NA | NA | NA | NA | NA | NA | NA |
| Unexposed | NA | NA | NA | NA | NA | NA | NA | NA |
| Combat mTBI | -0.051 | 0.036 | -1.395 | 0.164 | -0.037 | 0.048 | -0.779 | 0.437 |
| Non-Combat TBI | -0.019 | 0.040 | -0.481 | 0.631 | -0.012 | 0.042 | -0.279 | 0.780 |
| NUMBER OF BLAST PCES | -0.007 | 0.007 | -1.070 | 0.286 | -0.004 | 0.008 | -0.514 | 0.608 |
| Number of Positive mTBIs | -0.005 | 0.008 | -0.648 | 0.517 | 0.016 | 0.011 | 1.527 | 0.128 |
| Number of TBI with PTA+LOC | -0.085 | 0.023 | -3.638 | 0.000 | -0.089 | 0.027 | -3.355 | 0.001 |

1. **Central Midline Delta**

|  | Unadjusted | | | | Adjusted | | | |
| --- | --- | --- | --- | --- | --- | --- | --- | --- |
| Measure | Est. | SE | t | p | Est. | SE | t | p |
| DRRI2 | 0.027 | 0.013 | 2.056 | 0.041 | 0.003 | 0.018 | 0.182 | 0.855 |
| TBIQOL Cognition | -0.036 | 0.019 | -1.928 | 0.055 | 0.022 | 0.037 | 0.585 | 0.559 |
| TBIQOL Executive Function | -0.056 | 0.023 | -2.420 | 0.016 | -0.006 | 0.046 | -0.130 | 0.896 |
| Fluid Cognition | -0.053 | 0.015 | -3.462 | 0.001 | -0.034 | 0.019 | -1.782 | 0.076 |
| TOPF | -0.066 | 0.016 | -4.155 | 0.000 | -0.043 | 0.018 | -2.411 | 0.017 |
| AGE | -0.018 | 0.019 | -0.956 | 0.340 | -0.040 | 0.022 | -1.782 | 0.076 |
| PCL5 | 0.047 | 0.009 | 5.051 | 0.000 | 0.062 | 0.019 | 3.162 | 0.002 |
| PHQ9 | 0.091 | 0.030 | 2.993 | 0.003 | -0.082 | 0.063 | -1.295 | 0.196 |
| TBI Exposure | NA | NA | NA | NA | NA | NA | NA | NA |
| Unexposed | NA | NA | NA | NA | NA | NA | NA | NA |
| Combat mTBI | 0.502 | 0.472 | 1.065 | 0.288 | 0.082 | 0.668 | 0.123 | 0.902 |
| Non-Combat TBI | -0.207 | 0.519 | -0.400 | 0.689 | -0.016 | 0.583 | -0.027 | 0.978 |
| NUMBER OF BLAST PCES | 0.035 | 0.086 | 0.407 | 0.684 | 0.016 | 0.105 | 0.155 | 0.877 |
| Number of Positive mTBIs | 0.046 | 0.105 | 0.438 | 0.662 | -0.054 | 0.148 | -0.365 | 0.716 |
| Number of TBI with PTA+LOC | 0.510 | 0.311 | 1.641 | 0.102 | 0.104 | 0.371 | 0.280 | 0.779 |

1. **Central Midline Gamma**

|  | Unadjusted | | | | Adjusted | | | |
| --- | --- | --- | --- | --- | --- | --- | --- | --- |
| Measure | Est. | SE | t | p | Est. | SE | t | p |
| DRRI2 | 0.000 | 0.001 | -0.774 | 0.439 | 0.000 | 0.001 | 0.274 | 0.784 |
| TBIQOL Cognition | 0.001 | 0.001 | 0.962 | 0.337 | -0.001 | 0.002 | -0.759 | 0.448 |
| TBIQOL Executive Function | 0.002 | 0.001 | 1.490 | 0.137 | 0.002 | 0.002 | 1.087 | 0.278 |
| Fluid Cognition | 0.001 | 0.001 | 1.645 | 0.101 | 0.001 | 0.001 | 1.005 | 0.316 |
| TOPF | 0.001 | 0.001 | 1.870 | 0.062 | 0.001 | 0.001 | 1.426 | 0.155 |
| AGE | 0.000 | 0.001 | 0.074 | 0.941 | 0.001 | 0.001 | 0.530 | 0.596 |
| PCL5 | -0.001 | 0.000 | -1.211 | 0.227 | 0.000 | 0.001 | 0.100 | 0.920 |
| PHQ9 | -0.002 | 0.001 | -1.132 | 0.258 | 0.000 | 0.003 | 0.014 | 0.988 |
| TBI Exposure | NA | NA | NA | NA | NA | NA | NA | NA |
| Unexposed | NA | NA | NA | NA | NA | NA | NA | NA |
| Combat mTBI | -0.028 | 0.020 | -1.363 | 0.174 | -0.032 | 0.032 | -1.027 | 0.305 |
| Non-Combat TBI | -0.028 | 0.022 | -1.256 | 0.210 | -0.037 | 0.028 | -1.352 | 0.177 |
| NUMBER OF BLAST PCES | -0.004 | 0.004 | -1.194 | 0.233 | -0.006 | 0.005 | -1.243 | 0.215 |
| Number of Positive mTBIs | -0.003 | 0.005 | -0.682 | 0.496 | 0.004 | 0.007 | 0.589 | 0.556 |
| Number of TBI with PTA+LOC | -0.009 | 0.013 | -0.636 | 0.525 | 0.001 | 0.018 | 0.074 | 0.941 |

1. **Central Midline Theta**

|  | Unadjusted | | | | Adjusted | | | |
| --- | --- | --- | --- | --- | --- | --- | --- | --- |
| Measure | Est. | SE | t | p | Est. | SE | t | p |
| DRRI2 | 0.017 | 0.010 | 1.804 | 0.072 | 0.017 | 0.012 | 1.361 | 0.175 |
| TBIQOL Cognition | -0.021 | 0.013 | -1.600 | 0.111 | 0.000 | 0.026 | 0.013 | 0.989 |
| TBIQOL Executive Function | -0.024 | 0.016 | -1.460 | 0.145 | -0.021 | 0.032 | -0.675 | 0.500 |
| Fluid Cognition | -0.021 | 0.011 | -1.953 | 0.052 | 0.000 | 0.013 | -0.017 | 0.986 |
| TOPF | -0.022 | 0.012 | -1.819 | 0.070 | -0.019 | 0.012 | -1.551 | 0.122 |
| AGE | -0.005 | 0.014 | -0.358 | 0.721 | -0.002 | 0.015 | -0.108 | 0.914 |
| PCL5 | 0.015 | 0.007 | 2.096 | 0.037 | 0.033 | 0.013 | 2.475 | 0.014 |
| PHQ9 | 0.025 | 0.023 | 1.086 | 0.278 | -0.083 | 0.043 | -1.921 | 0.056 |
| TBI Exposure | NA | NA | NA | NA | NA | NA | NA | NA |
| Unexposed | NA | NA | NA | NA | NA | NA | NA | NA |
| Combat mTBI | 0.068 | 0.351 | 0.194 | 0.846 | -0.082 | 0.457 | -0.180 | 0.857 |
| Non-Combat TBI | 0.275 | 0.386 | 0.713 | 0.476 | 0.076 | 0.398 | 0.190 | 0.850 |
| NUMBER OF BLAST PCES | -0.093 | 0.064 | -1.457 | 0.146 | -0.080 | 0.072 | -1.111 | 0.267 |
| Number of Positive mTBIs | -0.059 | 0.078 | -0.755 | 0.451 | -0.060 | 0.101 | -0.596 | 0.552 |
| Number of TBI with PTA+LOC | -0.017 | 0.231 | -0.073 | 0.942 | -0.223 | 0.254 | -0.877 | 0.381 |

1. **Central Right Alpha**

|  | Unadjusted | | | | Adjusted | | | |
| --- | --- | --- | --- | --- | --- | --- | --- | --- |
| Measure | Est. | SE | t | p | Est. | SE | t | p |
| DRRI2 | -0.005 | 0.008 | -0.564 | 0.573 | 0.006 | 0.012 | 0.482 | 0.630 |
| TBIQOL Cognition | 0.009 | 0.012 | 0.788 | 0.431 | 0.013 | 0.024 | 0.533 | 0.594 |
| TBIQOL Executive Function | -0.001 | 0.015 | -0.076 | 0.939 | -0.029 | 0.030 | -0.963 | 0.336 |
| Fluid Cognition | -0.004 | 0.010 | -0.369 | 0.713 | -0.005 | 0.012 | -0.386 | 0.700 |
| TOPF | 0.008 | 0.010 | 0.755 | 0.451 | 0.012 | 0.012 | 1.015 | 0.311 |
| AGE | 0.003 | 0.012 | 0.282 | 0.778 | -0.006 | 0.015 | -0.425 | 0.671 |
| PCL5 | 0.001 | 0.006 | 0.187 | 0.852 | 0.038 | 0.013 | 2.985 | 0.003 |
| PHQ9 | -0.023 | 0.019 | -1.160 | 0.247 | -0.133 | 0.041 | -3.228 | 0.001 |
| TBI Exposure | NA | NA | NA | NA | NA | NA | NA | NA |
| Unexposed | NA | NA | NA | NA | NA | NA | NA | NA |
| Combat mTBI | -0.461 | 0.299 | -1.543 | 0.124 | -0.881 | 0.438 | -2.014 | 0.045 |
| Non-Combat TBI | -0.070 | 0.328 | -0.214 | 0.831 | -0.216 | 0.382 | -0.565 | 0.573 |
| NUMBER OF BLAST PCES | -0.070 | 0.054 | -1.287 | 0.199 | -0.064 | 0.069 | -0.925 | 0.356 |
| Number of Positive mTBIs | -0.020 | 0.067 | -0.295 | 0.768 | 0.144 | 0.097 | 1.482 | 0.139 |
| Number of TBI with PTA+LOC | -0.274 | 0.197 | -1.390 | 0.166 | -0.550 | 0.243 | -2.261 | 0.024 |

|  | Unadjusted | | | | Adjusted | | | |
| --- | --- | --- | --- | --- | --- | --- | --- | --- |
| Measure | Est. | SE | t | p | Est. | SE | t | p |
| DRRI2 | -0.002 | 0.001 | -2.627 | 0.009 | -0.002 | 0.001 | -2.077 | 0.039 |
| TBIQOL Cognition | 0.000 | 0.001 | 0.064 | 0.949 | -0.004 | 0.003 | -1.662 | 0.098 |
| TBIQOL Executive Function | 0.001 | 0.002 | 0.640 | 0.522 | 0.002 | 0.003 | 0.794 | 0.428 |
| Fluid Cognition | 0.002 | 0.001 | 1.963 | 0.051 | 0.003 | 0.001 | 2.530 | 0.012 |
| TOPF | 0.000 | 0.001 | 0.212 | 0.832 | -0.001 | 0.001 | -1.104 | 0.270 |
| AGE | 0.002 | 0.001 | 1.301 | 0.194 | 0.003 | 0.002 | 1.769 | 0.078 |
| PCL5 | 0.000 | 0.001 | -0.597 | 0.551 | 0.001 | 0.001 | 1.045 | 0.297 |
| PHQ9 | -0.003 | 0.002 | -1.470 | 0.143 | -0.006 | 0.004 | -1.406 | 0.161 |
| TBI Exposure | NA | NA | NA | NA | NA | NA | NA | NA |
| Unexposed | NA | NA | NA | NA | NA | NA | NA | NA |
| Combat mTBI | -0.043 | 0.033 | -1.308 | 0.192 | -0.039 | 0.045 | -0.861 | 0.390 |
| Non-Combat TBI | -0.006 | 0.036 | -0.154 | 0.878 | -0.028 | 0.039 | -0.700 | 0.485 |
| NUMBER OF BLAST PCES | -0.009 | 0.006 | -1.450 | 0.148 | -0.004 | 0.007 | -0.599 | 0.550 |
| Number of Positive mTBIs | 0.002 | 0.007 | 0.274 | 0.784 | 0.028 | 0.010 | 2.780 | 0.006 |
| Number of TBI with PTA+LOC | -0.080 | 0.021 | -3.712 | 0.000 | -0.100 | 0.025 | -3.987 | 0.000 |

**AA. Central Right Beta**

1. **Central Right Delta**

|  | Unadjusted | | | | Adjusted | | | |
| --- | --- | --- | --- | --- | --- | --- | --- | --- |
| Measure | Est. | SE | t | p | Est. | SE | t | p |
| DRRI2 | 0.016 | 0.008 | 1.973 | 0.049 | 0.005 | 0.011 | 0.452 | 0.651 |
| TBIQOL Cognition | -0.022 | 0.012 | -1.870 | 0.062 | 0.019 | 0.023 | 0.816 | 0.415 |
| TBIQOL Executive Function | -0.038 | 0.014 | -2.638 | 0.009 | -0.013 | 0.029 | -0.449 | 0.654 |
| Fluid Cognition | -0.036 | 0.010 | -3.797 | 0.000 | -0.022 | 0.012 | -1.849 | 0.065 |
| TOPF | -0.044 | 0.010 | -4.379 | 0.000 | -0.028 | 0.011 | -2.552 | 0.011 |
| AGE | -0.010 | 0.012 | -0.811 | 0.418 | -0.023 | 0.014 | -1.611 | 0.108 |
| PCL5 | 0.032 | 0.006 | 5.338 | 0.000 | 0.043 | 0.012 | 3.553 | 0.000 |
| PHQ9 | 0.058 | 0.019 | 3.016 | 0.003 | -0.065 | 0.039 | -1.660 | 0.098 |
| TBI Exposure | NA | NA | NA | NA | NA | NA | NA | NA |
| Unexposed | NA | NA | NA | NA | NA | NA | NA | NA |
| Combat mTBI | 0.274 | 0.299 | 0.916 | 0.360 | -0.044 | 0.416 | -0.106 | 0.916 |
| Non-Combat TBI | -0.063 | 0.329 | -0.191 | 0.848 | 0.029 | 0.363 | 0.081 | 0.936 |
| NUMBER OF BLAST PCES | -0.001 | 0.055 | -0.012 | 0.991 | -0.010 | 0.065 | -0.158 | 0.875 |
| Number of Positive mTBIs | 0.036 | 0.067 | 0.546 | 0.586 | -0.017 | 0.092 | -0.182 | 0.856 |
| Number of TBI with PTA+LOC | 0.338 | 0.197 | 1.720 | 0.086 | 0.058 | 0.231 | 0.250 | 0.803 |

1. **Central Right Gamma**

|  | Unadjusted | | | | Adjusted | | | |
| --- | --- | --- | --- | --- | --- | --- | --- | --- |
| Measure | Est. | SE | t | p | Est. | SE | t | p |
| DRRI2 | 0.000 | 0.000 | -0.225 | 0.822 | 0.000 | 0.000 | -0.722 | 0.471 |
| TBIQOL Cognition | 0.000 | 0.000 | -1.215 | 0.225 | 0.000 | 0.000 | -0.900 | 0.369 |
| TBIQOL Executive Function | 0.000 | 0.000 | -0.288 | 0.774 | 0.001 | 0.001 | 1.601 | 0.111 |
| Fluid Cognition | 0.000 | 0.000 | -0.611 | 0.541 | 0.000 | 0.000 | -0.229 | 0.819 |
| TOPF | 0.000 | 0.000 | 0.691 | 0.490 | 0.000 | 0.000 | 0.925 | 0.356 |
| AGE | 0.000 | 0.000 | 1.415 | 0.158 | 0.000 | 0.000 | 1.105 | 0.270 |
| PCL5 | 0.000 | 0.000 | 0.472 | 0.637 | 0.000 | 0.000 | -0.325 | 0.746 |
| PHQ9 | 0.000 | 0.000 | 0.862 | 0.389 | 0.001 | 0.001 | 1.055 | 0.292 |
| TBI Exposure | NA | NA | NA | NA | NA | NA | NA | NA |
| Unexposed | NA | NA | NA | NA | NA | NA | NA | NA |
| Combat mTBI | 0.007 | 0.005 | 1.475 | 0.141 | 0.002 | 0.007 | 0.250 | 0.803 |
| Non-Combat TBI | 0.009 | 0.005 | 1.668 | 0.096 | 0.002 | 0.006 | 0.371 | 0.711 |
| NUMBER OF BLAST PCES | 0.001 | 0.001 | 1.329 | 0.185 | 0.001 | 0.001 | 0.761 | 0.448 |
| Number of Positive mTBIs | 0.003 | 0.001 | 2.859 | 0.005 | 0.004 | 0.002 | 2.738 | 0.007 |
| Number of TBI with PTA+LOC | -0.004 | 0.003 | -1.095 | 0.274 | -0.009 | 0.004 | -2.255 | 0.025 |

|  | Unadjusted | | | | Adjusted | | | |
| --- | --- | --- | --- | --- | --- | --- | --- | --- |
| Measure | Est. | SE | t | p | Est. | SE | t | p |
| DRRI2 | 0.009 | 0.007 | 1.314 | 0.190 | 0.012 | 0.009 | 1.402 | 0.162 |
| TBIQOL Cognition | -0.011 | 0.009 | -1.236 | 0.218 | 0.001 | 0.018 | 0.027 | 0.978 |
| TBIQOL Executive Function | -0.015 | 0.011 | -1.311 | 0.191 | -0.019 | 0.023 | -0.858 | 0.392 |
| Fluid Cognition | -0.012 | 0.008 | -1.628 | 0.105 | 0.002 | 0.009 | 0.205 | 0.838 |
| TOPF | -0.013 | 0.008 | -1.621 | 0.106 | -0.012 | 0.009 | -1.339 | 0.182 |
| AGE | -0.001 | 0.010 | -0.084 | 0.933 | 0.001 | 0.011 | 0.097 | 0.923 |
| PCL5 | 0.009 | 0.005 | 1.787 | 0.075 | 0.024 | 0.010 | 2.483 | 0.014 |
| PHQ9 | 0.011 | 0.016 | 0.720 | 0.472 | -0.068 | 0.031 | -2.196 | 0.029 |
| TBI Exposure | NA | NA | NA | NA | NA | NA | NA | NA |
| Unexposed | NA | NA | NA | NA | NA | NA | NA | NA |
| Combat mTBI | -0.042 | 0.243 | -0.174 | 0.862 | -0.146 | 0.327 | -0.447 | 0.655 |
| Non-Combat TBI | 0.118 | 0.267 | 0.444 | 0.658 | -0.001 | 0.286 | -0.004 | 0.997 |
| NUMBER OF BLAST PCES | -0.079 | 0.044 | -1.798 | 0.073 | -0.070 | 0.051 | -1.357 | 0.176 |
| Number of Positive mTBIs | -0.053 | 0.054 | -0.976 | 0.330 | -0.041 | 0.073 | -0.564 | 0.573 |
| Number of TBI with PTA+LOC | -0.051 | 0.160 | -0.317 | 0.751 | -0.155 | 0.182 | -0.853 | 0.394 |

1. **Central Right Theta**
2. **Posterior Left Alpha**

|  | Unadjusted | | | | Adjusted | | | |
| --- | --- | --- | --- | --- | --- | --- | --- | --- |
| Measure | Est. | SE | t | p | Est. | SE | t | p |
| DRRI2 | -0.009 | 0.011 | -0.852 | 0.395 | 0.004 | 0.015 | 0.305 | 0.760 |
| TBIQOL Cognition | -0.001 | 0.015 | -0.077 | 0.938 | 0.023 | 0.031 | 0.749 | 0.454 |
| TBIQOL Executive Function | -0.019 | 0.019 | -1.012 | 0.312 | -0.083 | 0.038 | -2.177 | 0.030 |
| Fluid Cognition | 0.002 | 0.013 | 0.142 | 0.887 | -0.011 | 0.016 | -0.715 | 0.475 |
| TOPF | 0.023 | 0.013 | 1.735 | 0.084 | 0.026 | 0.015 | 1.774 | 0.077 |
| AGE | -0.014 | 0.015 | -0.930 | 0.353 | -0.032 | 0.018 | -1.743 | 0.082 |
| PCL5 | 0.000 | 0.008 | -0.061 | 0.952 | 0.041 | 0.016 | 2.553 | 0.011 |
| PHQ9 | -0.025 | 0.025 | -1.002 | 0.317 | -0.175 | 0.052 | -3.359 | 0.001 |
| TBI Exposure | NA | NA | NA | NA | NA | NA | NA | NA |
| Unexposed | NA | NA | NA | NA | NA | NA | NA | NA |
| Combat mTBI | -0.444 | 0.379 | -1.171 | 0.243 | -1.170 | 0.551 | -2.126 | 0.034 |
| Non-Combat TBI | 0.495 | 0.417 | 1.189 | 0.235 | 0.225 | 0.480 | 0.468 | 0.640 |
| NUMBER OF BLAST PCES | -0.133 | 0.069 | -1.918 | 0.056 | -0.150 | 0.086 | -1.738 | 0.083 |
| Number of Positive mTBIs | 0.028 | 0.085 | 0.329 | 0.742 | 0.269 | 0.122 | 2.203 | 0.028 |
| Number of TBI with PTA+LOC | -0.398 | 0.251 | -1.585 | 0.114 | -0.761 | 0.306 | -2.487 | 0.013 |

1. **Posterior Left Beta**

|  | Single | | | | Adjusted | | | |
| --- | --- | --- | --- | --- | --- | --- | --- | --- |
| Measure | Est. | SE | t | p | Est. | SE | t | p |
| DRRI2 | -0.003 | 0.001 | -3.235 | 0.001 | -0.003 | 0.001 | -2.403 | 0.017 |
| TBIQOL Cognition | 0.000 | 0.001 | -0.009 | 0.993 | -0.005 | 0.002 | -2.152 | 0.032 |
| TBIQOL Executive Function | 0.001 | 0.002 | 0.698 | 0.486 | 0.001 | 0.003 | 0.492 | 0.623 |
| Fluid Cognition | 0.004 | 0.001 | 3.809 | 0.000 | 0.003 | 0.001 | 2.782 | 0.006 |
| TOPF | 0.002 | 0.001 | 1.944 | 0.053 | 0.000 | 0.001 | 0.058 | 0.954 |
| AGE | -0.001 | 0.001 | -1.105 | 0.270 | -0.001 | 0.001 | -0.358 | 0.721 |
| PCL5 | -0.001 | 0.001 | -2.133 | 0.034 | 0.000 | 0.001 | 0.001 | 0.999 |
| PHQ9 | -0.004 | 0.002 | -2.008 | 0.046 | -0.004 | 0.004 | -1.044 | 0.298 |
| TBI Exposure | NA | NA | NA | NA | NA | NA | NA | NA |
| Unexposed | NA | NA | NA | NA | NA | NA | NA | NA |
| Combat mTBI | -0.052 | 0.031 | -1.687 | 0.093 | -0.012 | 0.043 | -0.272 | 0.786 |
| Non-Combat TBI | -0.013 | 0.034 | -0.392 | 0.695 | -0.015 | 0.038 | -0.407 | 0.684 |
| NUMBER OF BLAST PCES | -0.014 | 0.006 | -2.557 | 0.011 | -0.010 | 0.007 | -1.542 | 0.124 |
| Number of Positive mTBIs | -0.006 | 0.007 | -0.844 | 0.399 | 0.016 | 0.010 | 1.724 | 0.086 |
| Number of TBI with PTA+LOC | -0.076 | 0.020 | -3.845 | 0.000 | -0.083 | 0.024 | -3.476 | 0.001 |

1. **Posterior Left Delta**

|  | Unadjusted | | | | Adjusted | | | |
| --- | --- | --- | --- | --- | --- | --- | --- | --- |
| Measure | Est. | SE | t | p | Est. | SE | t | p |
| DRRI2 | 0.011 | 0.005 | 2.045 | 0.042 | 0.007 | 0.007 | 1.011 | 0.313 |
| TBIQOL Cognition | -0.008 | 0.008 | -1.119 | 0.264 | 0.023 | 0.015 | 1.562 | 0.120 |
| TBIQOL Executive Function | -0.020 | 0.009 | -2.097 | 0.037 | -0.018 | 0.018 | -0.974 | 0.331 |
| Fluid Cognition | -0.012 | 0.006 | -1.962 | 0.051 | -0.003 | 0.007 | -0.410 | 0.682 |
| TOPF | -0.019 | 0.006 | -2.965 | 0.003 | -0.012 | 0.007 | -1.757 | 0.080 |
| AGE | -0.013 | 0.008 | -1.708 | 0.089 | -0.014 | 0.009 | -1.627 | 0.105 |
| PCL5 | 0.018 | 0.004 | 4.798 | 0.000 | 0.036 | 0.008 | 4.705 | 0.000 |
| PHQ9 | 0.027 | 0.012 | 2.144 | 0.033 | -0.063 | 0.025 | -2.528 | 0.012 |
| TBI Exposure | NA | NA | NA | NA | NA | NA | NA | NA |
| Unexposed | NA | NA | NA | NA | NA | NA | NA | NA |
| Combat mTBI | 0.126 | 0.191 | 0.659 | 0.511 | -0.103 | 0.265 | -0.388 | 0.699 |
| Non-Combat TBI | -0.042 | 0.210 | -0.199 | 0.842 | -0.009 | 0.231 | -0.037 | 0.970 |
| NUMBER OF BLAST PCES | -0.045 | 0.035 | -1.301 | 0.194 | -0.060 | 0.042 | -1.451 | 0.148 |
| Number of Positive mTBIs | 0.022 | 0.043 | 0.520 | 0.603 | 0.047 | 0.059 | 0.809 | 0.419 |
| Number of TBI with PTA+LOC | 0.104 | 0.126 | 0.826 | 0.409 | -0.135 | 0.147 | -0.919 | 0.359 |

1. **Posterior Left Gamma**

|  | Unadjusted | | | | Adjusted | | | |
| --- | --- | --- | --- | --- | --- | --- | --- | --- |
| Measure | Est. | SE | t | p | Est. | SE | t | p |
| DRRI2 | 0.000 | 0.000 | 0.199 | 0.842 | 0.000 | 0.000 | -1.259 | 0.209 |
| TBIQOL Cognition | 0.000 | 0.000 | -1.745 | 0.082 | -0.001 | 0.000 | -1.814 | 0.071 |
| TBIQOL Executive Function | 0.000 | 0.000 | -0.462 | 0.645 | 0.001 | 0.000 | 1.656 | 0.099 |
| Fluid Cognition | 0.000 | 0.000 | -0.178 | 0.859 | 0.000 | 0.000 | -0.770 | 0.442 |
| TOPF | 0.000 | 0.000 | 0.832 | 0.406 | 0.000 | 0.000 | 0.596 | 0.552 |
| AGE | 0.000 | 0.000 | -0.994 | 0.321 | 0.000 | 0.000 | -1.195 | 0.233 |
| PCL5 | 0.000 | 0.000 | 0.307 | 0.759 | 0.000 | 0.000 | -0.643 | 0.521 |
| PHQ9 | 0.000 | 0.000 | 0.667 | 0.505 | 0.000 | 0.001 | 0.659 | 0.510 |
| TBI Exposure | NA | NA | NA | NA | NA | NA | NA | NA |
| Unexposed | NA | NA | NA | NA | NA | NA | NA | NA |
| Combat mTBI | 0.008 | 0.004 | 2.074 | 0.039 | 0.009 | 0.006 | 1.564 | 0.119 |
| Non-Combat TBI | 0.005 | 0.004 | 1.180 | 0.239 | 0.004 | 0.005 | 0.734 | 0.464 |
| NUMBER OF BLAST PCES | 0.001 | 0.001 | 0.991 | 0.322 | 0.001 | 0.001 | 0.666 | 0.506 |
| Number of Positive mTBIs | 0.002 | 0.001 | 1.732 | 0.084 | 0.001 | 0.001 | 0.916 | 0.361 |
| Number of TBI with PTA+LOC | -0.002 | 0.003 | -0.933 | 0.352 | -0.007 | 0.003 | -2.018 | 0.044 |

1. **Posterior Left Theta**

|  | Unadjusted | | | | Adjusted | | | |
| --- | --- | --- | --- | --- | --- | --- | --- | --- |
| Measure | Est. | SE | t | p | Est. | SE | t | p |
| DRRI2 | 0.004 | 0.005 | 0.936 | 0.350 | 0.009 | 0.006 | 1.373 | 0.171 |
| TBIQOL Cognition | -0.005 | 0.007 | -0.770 | 0.442 | -0.003 | 0.013 | -0.227 | 0.820 |
| TBIQOL Executive Function | -0.006 | 0.008 | -0.725 | 0.469 | -0.009 | 0.016 | -0.527 | 0.599 |
| Fluid Cognition | -0.003 | 0.005 | -0.544 | 0.587 | 0.008 | 0.007 | 1.167 | 0.244 |
| TOPF | -0.007 | 0.006 | -1.113 | 0.266 | -0.008 | 0.006 | -1.266 | 0.206 |
| AGE | -0.001 | 0.007 | -0.140 | 0.889 | 0.003 | 0.008 | 0.440 | 0.660 |
| PCL5 | 0.004 | 0.004 | 1.171 | 0.243 | 0.016 | 0.007 | 2.325 | 0.021 |
| PHQ9 | 0.002 | 0.011 | 0.173 | 0.863 | -0.047 | 0.022 | -2.136 | 0.034 |
| TBI Exposure | NA | NA | NA | NA | NA | NA | NA | NA |
| Unexposed | NA | NA | NA | NA | NA | NA | NA | NA |
| Combat mTBI | -0.062 | 0.172 | -0.361 | 0.719 | -0.104 | 0.234 | -0.445 | 0.656 |
| Non-Combat TBI | 0.089 | 0.189 | 0.470 | 0.639 | 0.008 | 0.204 | 0.038 | 0.970 |
| NUMBER OF BLAST PCES | -0.084 | 0.031 | -2.698 | 0.007 | -0.082 | 0.037 | -2.247 | 0.025 |
| Number of Positive mTBIs | -0.037 | 0.038 | -0.968 | 0.334 | 0.000 | 0.052 | -0.003 | 0.998 |
| Number of TBI with PTA+LOC | -0.063 | 0.113 | -0.559 | 0.577 | -0.161 | 0.130 | -1.238 | 0.217 |

1. **Posterior Midline Alpha**

|  | Unadjusted | | | | Adjusted | | | |
| --- | --- | --- | --- | --- | --- | --- | --- | --- |
| Measure | Est. | SE | t | p | Est. | SE | t | p |
| DRRI2 | -0.006 | 0.012 | -0.466 | 0.641 | 0.005 | 0.017 | 0.306 | 0.760 |
| TBIQOL Cognition | -0.007 | 0.017 | -0.411 | 0.681 | 0.033 | 0.035 | 0.955 | 0.341 |
| TBIQOL Executive Function | -0.026 | 0.022 | -1.207 | 0.228 | -0.098 | 0.043 | -2.257 | 0.025 |
| Fluid Cognition | 0.003 | 0.014 | 0.202 | 0.840 | -0.011 | 0.018 | -0.652 | 0.515 |
| TOPF | 0.028 | 0.015 | 1.896 | 0.059 | 0.034 | 0.017 | 2.053 | 0.041 |
| AGE | -0.018 | 0.018 | -1.011 | 0.313 | -0.038 | 0.021 | -1.797 | 0.073 |
| PCL5 | 0.005 | 0.009 | 0.580 | 0.562 | 0.059 | 0.018 | 3.250 | 0.001 |
| PHQ9 | -0.020 | 0.029 | -0.715 | 0.475 | -0.214 | 0.059 | -3.621 | 0.000 |
| TBI Exposure | NA | NA | NA | NA | NA | NA | NA | NA |
| Unexposed | NA | NA | NA | NA | NA | NA | NA | NA |
| Combat mTBI | -0.388 | 0.437 | -0.887 | 0.376 | -1.420 | 0.625 | -2.272 | 0.024 |
| Non-Combat TBI | 0.523 | 0.480 | 1.089 | 0.277 | 0.136 | 0.545 | 0.250 | 0.803 |
| NUMBER OF BLAST PCES | -0.139 | 0.080 | -1.749 | 0.081 | -0.186 | 0.098 | -1.892 | 0.059 |
| Number of Positive mTBIs | 0.074 | 0.098 | 0.755 | 0.451 | 0.359 | 0.139 | 2.587 | 0.010 |
| Number of TBI with PTA+LOC | -0.501 | 0.288 | -1.735 | 0.084 | -0.907 | 0.347 | -2.612 | 0.009 |

1. **Posterior Midline Beta**

|  | Unadjusted | | | | Adjusted | | | |
| --- | --- | --- | --- | --- | --- | --- | --- | --- |
| Measure | Est. | SE | t | p | Est. | SE | t | p |
| DRRI2 | -0.003 | 0.001 | -3.106 | 0.002 | -0.002 | 0.001 | -1.966 | 0.050 |
| TBIQOL Cognition | 0.000 | 0.001 | 0.250 | 0.803 | -0.005 | 0.002 | -2.035 | 0.043 |
| TBIQOL Executive Function | 0.001 | 0.001 | 0.864 | 0.388 | 0.001 | 0.003 | 0.243 | 0.808 |
| Fluid Cognition | 0.004 | 0.001 | 4.281 | 0.000 | 0.004 | 0.001 | 3.425 | 0.001 |
| TOPF | 0.002 | 0.001 | 1.560 | 0.120 | -0.001 | 0.001 | -0.500 | 0.617 |
| AGE | -0.001 | 0.001 | -1.185 | 0.237 | 0.000 | 0.001 | -0.294 | 0.769 |
| PCL5 | -0.001 | 0.001 | -2.239 | 0.026 | 0.001 | 0.001 | 0.585 | 0.559 |
| PHQ9 | -0.005 | 0.002 | -2.416 | 0.016 | -0.007 | 0.004 | -1.785 | 0.075 |
| TBI Exposure | NA | NA | NA | NA | NA | NA | NA | NA |
| Unexposed | NA | NA | NA | NA | NA | NA | NA | NA |
| Combat mTBI | -0.060 | 0.030 | -2.000 | 0.046 | -0.030 | 0.042 | -0.725 | 0.469 |
| Non-Combat TBI | -0.015 | 0.033 | -0.468 | 0.640 | -0.015 | 0.037 | -0.401 | 0.689 |
| NUMBER OF BLAST PCES | -0.014 | 0.005 | -2.659 | 0.008 | -0.011 | 0.007 | -1.727 | 0.085 |
| Number of Positive mTBIs | -0.007 | 0.007 | -1.054 | 0.293 | 0.016 | 0.009 | 1.747 | 0.082 |
| Number of TBI with PTA+LOC | -0.076 | 0.019 | -3.914 | 0.000 | -0.075 | 0.023 | -3.219 | 0.001 |

1. **Posterior Midline Delta**

|  | Unadjusted | | | | Adjusted | | | |
| --- | --- | --- | --- | --- | --- | --- | --- | --- |
| Measure | Est. | SE | t | p | Est. | SE | t | p |
| DRRI2 | 0.011 | 0.006 | 1.767 | 0.078 | 0.005 | 0.008 | 0.584 | 0.560 |
| TBIQOL Cognition | -0.020 | 0.009 | -2.224 | 0.027 | 0.015 | 0.018 | 0.867 | 0.386 |
| TBIQOL Executive Function | -0.033 | 0.011 | -2.932 | 0.004 | -0.015 | 0.022 | -0.673 | 0.502 |
| Fluid Cognition | -0.020 | 0.007 | -2.759 | 0.006 | -0.009 | 0.009 | -0.965 | 0.335 |
| TOPF | -0.022 | 0.008 | -2.871 | 0.004 | -0.010 | 0.008 | -1.242 | 0.215 |
| AGE | -0.014 | 0.009 | -1.474 | 0.142 | -0.019 | 0.011 | -1.746 | 0.082 |
| PCL5 | 0.026 | 0.004 | 5.813 | 0.000 | 0.043 | 0.009 | 4.613 | 0.000 |
| PHQ9 | 0.047 | 0.015 | 3.221 | 0.001 | -0.066 | 0.030 | -2.212 | 0.028 |
| TBI Exposure | NA | NA | NA | NA | NA | NA | NA | NA |
| Unexposed | NA | NA | NA | NA | NA | NA | NA | NA |
| Combat mTBI | 0.152 | 0.229 | 0.667 | 0.505 | -0.102 | 0.316 | -0.324 | 0.746 |
| Non-Combat TBI | -0.008 | 0.251 | -0.033 | 0.974 | 0.032 | 0.276 | 0.115 | 0.909 |
| NUMBER OF BLAST PCES | -0.055 | 0.042 | -1.318 | 0.189 | -0.070 | 0.050 | -1.417 | 0.157 |
| Number of Positive mTBIs | 0.009 | 0.051 | 0.168 | 0.866 | -0.003 | 0.070 | -0.047 | 0.963 |
| Number of TBI with PTA+LOC | 0.211 | 0.150 | 1.404 | 0.161 | 0.002 | 0.176 | 0.011 | 0.991 |

1. **Posterior Midline Gamma**

|  | Unadjusted | | | | Adjusted | | | |
| --- | --- | --- | --- | --- | --- | --- | --- | --- |
| Measure | Est. | SE | t | p | Est. | SE | t | p |
| DRRI2 | 0.000 | 0.000 | -0.726 | 0.468 | 0.000 | 0.000 | -1.259 | 0.209 |
| TBIQOL Cognition | 0.000 | 0.000 | -0.412 | 0.681 | 0.000 | 0.000 | -1.565 | 0.119 |
| TBIQOL Executive Function | 0.000 | 0.000 | 0.776 | 0.439 | 0.001 | 0.000 | 2.171 | 0.031 |
| Fluid Cognition | 0.000 | 0.000 | -0.844 | 0.399 | 0.000 | 0.000 | -1.624 | 0.106 |
| TOPF | 0.000 | 0.000 | 1.135 | 0.257 | 0.000 | 0.000 | 1.422 | 0.156 |
| AGE | 0.000 | 0.000 | -0.393 | 0.695 | 0.000 | 0.000 | -1.229 | 0.220 |
| PCL5 | 0.000 | 0.000 | -0.243 | 0.808 | 0.000 | 0.000 | -0.561 | 0.575 |
| PHQ9 | 0.000 | 0.000 | 0.074 | 0.941 | 0.000 | 0.001 | 0.819 | 0.413 |
| TBI Exposure | NA | NA | NA | NA | NA | NA | NA | NA |
| Unexposed | NA | NA | NA | NA | NA | NA | NA | NA |
| Combat mTBI | 0.004 | 0.004 | 1.030 | 0.304 | 0.005 | 0.005 | 0.972 | 0.332 |
| Non-Combat TBI | 0.004 | 0.004 | 1.025 | 0.306 | 0.004 | 0.005 | 0.752 | 0.453 |
| NUMBER OF BLAST PCES | 0.000 | 0.001 | 0.294 | 0.769 | 0.000 | 0.001 | 0.450 | 0.653 |
| Number of Positive mTBIs | 0.000 | 0.001 | 0.559 | 0.577 | 0.000 | 0.001 | 0.161 | 0.872 |
| Number of TBI with PTA+LOC | 0.000 | 0.002 | -0.074 | 0.941 | -0.001 | 0.003 | -0.477 | 0.634 |

1. **Posterior Midline Theta**

|  | Unadjusted | | | | Adjusted | | | |
| --- | --- | --- | --- | --- | --- | --- | --- | --- |
| Measure | Est. | SE | t | p | Est. | SE | t | p |
| DRRI2 | 0.006 | 0.005 | 1.024 | 0.307 | 0.009 | 0.007 | 1.292 | 0.197 |
| TBIQOL Cognition | -0.010 | 0.008 | -1.339 | 0.182 | -0.003 | 0.015 | -0.225 | 0.822 |
| TBIQOL Executive Function | -0.013 | 0.009 | -1.360 | 0.175 | -0.011 | 0.019 | -0.579 | 0.563 |
| Fluid Cognition | -0.006 | 0.006 | -1.005 | 0.316 | 0.007 | 0.008 | 0.959 | 0.339 |
| TOPF | -0.011 | 0.007 | -1.546 | 0.123 | -0.010 | 0.007 | -1.332 | 0.184 |
| AGE | 0.000 | 0.008 | -0.020 | 0.984 | 0.003 | 0.009 | 0.377 | 0.707 |
| PCL5 | 0.008 | 0.004 | 2.066 | 0.040 | 0.021 | 0.008 | 2.653 | 0.008 |
| PHQ9 | 0.012 | 0.013 | 0.958 | 0.339 | -0.054 | 0.026 | -2.088 | 0.038 |
| TBI Exposure | NA | NA | NA | NA | NA | NA | NA | NA |
| Unexposed | NA | NA | NA | NA | NA | NA | NA | NA |
| Combat mTBI | -0.039 | 0.199 | -0.195 | 0.846 | -0.106 | 0.274 | -0.385 | 0.700 |
| Non-Combat TBI | 0.115 | 0.219 | 0.525 | 0.600 | 0.049 | 0.239 | 0.205 | 0.838 |
| NUMBER OF BLAST PCES | -0.095 | 0.036 | -2.645 | 0.009 | -0.097 | 0.043 | -2.249 | 0.025 |
| Number of Positive mTBIs | -0.043 | 0.044 | -0.964 | 0.336 | -0.016 | 0.061 | -0.264 | 0.792 |
| Number of TBI with PTA+LOC | -0.051 | 0.131 | -0.386 | 0.699 | -0.162 | 0.152 | -1.065 | 0.288 |

1. **Posterior Right Alpha**

|  | Unadjusted | | | | Adjusted | | | |
| --- | --- | --- | --- | --- | --- | --- | --- | --- |
| Measure | Est. | SE | t | p | Est. | SE | t | p |
| DRRI2 | -0.001 | 0.012 | -0.052 | 0.958 | 0.005 | 0.016 | 0.297 | 0.767 |
| TBIQOL Cognition | -0.008 | 0.017 | -0.500 | 0.617 | 0.035 | 0.034 | 1.002 | 0.317 |
| TBIQOL Executive Function | -0.029 | 0.021 | -1.373 | 0.171 | -0.086 | 0.043 | -2.015 | 0.045 |
| Fluid Cognition | -0.006 | 0.014 | -0.415 | 0.679 | -0.026 | 0.017 | -1.488 | 0.138 |
| TOPF | 0.024 | 0.015 | 1.619 | 0.107 | 0.031 | 0.016 | 1.930 | 0.055 |
| AGE | -0.024 | 0.017 | -1.384 | 0.167 | -0.051 | 0.021 | -2.474 | 0.014 |
| PCL5 | 0.006 | 0.009 | 0.709 | 0.479 | 0.043 | 0.018 | 2.378 | 0.018 |
| PHQ9 | -0.005 | 0.028 | -0.190 | 0.850 | -0.156 | 0.058 | -2.681 | 0.008 |
| TBI Exposure | NA | NA | NA | NA | NA | NA | NA | NA |
| Unexposed | NA | NA | NA | NA | NA | NA | NA | NA |
| Combat mTBI | -0.322 | 0.433 | -0.744 | 0.457 | -1.497 | 0.616 | -2.429 | 0.016 |
| Non-Combat TBI | 0.509 | 0.476 | 1.071 | 0.285 | 0.052 | 0.538 | 0.096 | 0.924 |
| NUMBER OF BLAST PCES | -0.093 | 0.079 | -1.174 | 0.241 | -0.159 | 0.097 | -1.643 | 0.101 |
| Number of Positive mTBIs | 0.111 | 0.097 | 1.144 | 0.253 | 0.396 | 0.137 | 2.896 | 0.004 |
| Number of TBI with PTA+LOC | -0.433 | 0.286 | -1.516 | 0.131 | -0.925 | 0.342 | -2.702 | 0.007 |

1. **Posterior Right Beta**

|  | Unadjusted | | | | Adjusted | | | |
| --- | --- | --- | --- | --- | --- | --- | --- | --- |
| Measure | Est. | SE | t | p | Est. | SE | t | p |
| DRRI2 | -0.002 | 0.001 | -3.078 | 0.002 | -0.002 | 0.001 | -1.738 | 0.083 |
| TBIQOL Cognition | 0.001 | 0.001 | 0.499 | 0.618 | -0.004 | 0.002 | -1.827 | 0.069 |
| TBIQOL Executive Function | 0.002 | 0.001 | 1.065 | 0.287 | 0.001 | 0.003 | 0.484 | 0.629 |
| Fluid Cognition | 0.004 | 0.001 | 3.891 | 0.000 | 0.003 | 0.001 | 2.893 | 0.004 |
| TOPF | 0.002 | 0.001 | 1.661 | 0.098 | 0.000 | 0.001 | -0.174 | 0.862 |
| AGE | -0.001 | 0.001 | -1.052 | 0.294 | -0.001 | 0.001 | -0.404 | 0.687 |
| PCL5 | -0.001 | 0.001 | -2.269 | 0.024 | 0.000 | 0.001 | 0.312 | 0.755 |
| PHQ9 | -0.004 | 0.002 | -2.224 | 0.027 | -0.005 | 0.004 | -1.314 | 0.190 |
| TBI Exposure | NA | NA | NA | NA | NA | NA | NA | NA |
| Unexposed | NA | NA | NA | NA | NA | NA | NA | NA |
| Combat mTBI | -0.055 | 0.029 | -1.884 | 0.060 | -0.029 | 0.042 | -0.688 | 0.492 |
| Non-Combat TBI | -0.004 | 0.032 | -0.135 | 0.893 | -0.004 | 0.036 | -0.107 | 0.915 |
| NUMBER OF BLAST PCES | -0.014 | 0.005 | -2.592 | 0.010 | -0.011 | 0.007 | -1.676 | 0.095 |
| Number of Positive mTBIs | -0.006 | 0.007 | -0.982 | 0.327 | 0.016 | 0.009 | 1.692 | 0.092 |
| Number of TBI with PTA+LOC | -0.070 | 0.019 | -3.682 | 0.000 | -0.072 | 0.023 | -3.102 | 0.002 |

1. **Posterior Right Delta**

|  | Unadjusted | | | | Adjusted | | | |
| --- | --- | --- | --- | --- | --- | --- | --- | --- |
| Measure | Est. | SE | t | p | Est. | SE | t | p |
| DRRI2 | 0.007 | 0.005 | 1.370 | 0.172 | 0.002 | 0.007 | 0.324 | 0.746 |
| TBIQOL Cognition | -0.011 | 0.007 | -1.443 | 0.150 | 0.021 | 0.015 | 1.461 | 0.145 |
| TBIQOL Executive Function | -0.023 | 0.009 | -2.519 | 0.012 | -0.019 | 0.018 | -1.046 | 0.297 |
| Fluid Cognition | -0.012 | 0.006 | -2.020 | 0.044 | -0.004 | 0.007 | -0.533 | 0.594 |
| TOPF | -0.017 | 0.006 | -2.739 | 0.007 | -0.009 | 0.007 | -1.289 | 0.199 |
| AGE | -0.012 | 0.008 | -1.626 | 0.105 | -0.016 | 0.009 | -1.840 | 0.067 |
| PCL5 | 0.019 | 0.004 | 5.090 | 0.000 | 0.033 | 0.008 | 4.291 | 0.000 |
| PHQ9 | 0.033 | 0.012 | 2.746 | 0.006 | -0.052 | 0.025 | -2.083 | 0.038 |
| TBI Exposure | NA | NA | NA | NA | NA | NA | NA | NA |
| Unexposed | NA | NA | NA | NA | NA | NA | NA | NA |
| Combat mTBI | 0.177 | 0.187 | 0.950 | 0.343 | -0.130 | 0.263 | -0.494 | 0.621 |
| Non-Combat TBI | 0.016 | 0.205 | 0.080 | 0.936 | -0.035 | 0.230 | -0.150 | 0.881 |
| NUMBER OF BLAST PCES | -0.059 | 0.034 | -1.747 | 0.082 | -0.072 | 0.041 | -1.752 | 0.081 |
| Number of Positive mTBIs | 0.034 | 0.042 | 0.816 | 0.415 | 0.030 | 0.058 | 0.521 | 0.602 |
| Number of TBI with PTA+LOC | 0.271 | 0.122 | 2.218 | 0.027 | 0.084 | 0.146 | 0.571 | 0.568 |

1. **Posterior Right Gamma**

|  | Unadjusted | | | | Adjusted | | | |
| --- | --- | --- | --- | --- | --- | --- | --- | --- |
| Measure | Est. | SE | t | p | Est. | SE | t | p |
| DRRI2 | 0.000 | 0.000 | -0.977 | 0.329 | 0.000 | 0.000 | -0.967 | 0.334 |
| TBIQOL Cognition | 0.000 | 0.000 | 0.208 | 0.836 | 0.000 | 0.000 | -1.771 | 0.078 |
| TBIQOL Executive Function | 0.000 | 0.000 | 1.581 | 0.115 | 0.001 | 0.000 | 2.006 | 0.046 |
| Fluid Cognition | 0.000 | 0.000 | 1.680 | 0.094 | 0.000 | 0.000 | 0.778 | 0.437 |
| TOPF | 0.000 | 0.000 | 2.085 | 0.038 | 0.000 | 0.000 | 1.304 | 0.193 |
| AGE | 0.000 | 0.000 | -0.073 | 0.942 | 0.000 | 0.000 | 0.178 | 0.859 |
| PCL5 | 0.000 | 0.000 | -1.625 | 0.105 | 0.000 | 0.000 | -0.329 | 0.743 |
| PHQ9 | 0.000 | 0.000 | -1.487 | 0.138 | 0.000 | 0.000 | -0.043 | 0.965 |
| TBI Exposure | NA | NA | NA | NA | NA | NA | NA | NA |
| Unexposed | NA | NA | NA | NA | NA | NA | NA | NA |
| Combat mTBI | 0.002 | 0.003 | 0.781 | 0.436 | 0.005 | 0.005 | 1.143 | 0.254 |
| Non-Combat TBI | -0.001 | 0.003 | -0.153 | 0.879 | -0.001 | 0.004 | -0.290 | 0.772 |
| NUMBER OF BLAST PCES | 0.000 | 0.001 | -0.471 | 0.638 | 0.000 | 0.001 | -0.518 | 0.605 |
| Number of Positive mTBIs | 0.000 | 0.001 | 0.660 | 0.510 | 0.001 | 0.001 | 0.979 | 0.329 |
| Number of TBI with PTA+LOC | -0.003 | 0.002 | -1.605 | 0.110 | -0.004 | 0.003 | -1.666 | 0.097 |

1. **Posterior Right Theta**

|  | Unadjusted | | | | Adjusted | | | |
| --- | --- | --- | --- | --- | --- | --- | --- | --- |
| Measure | Est. | SE | t | p | Est. | SE | t | p |
| DRRI2 | 0.004 | 0.004 | 0.850 | 0.396 | 0.007 | 0.006 | 1.163 | 0.246 |
| TBIQOL Cognition | -0.006 | 0.006 | -1.003 | 0.317 | 0.000 | 0.012 | -0.033 | 0.974 |
| TBIQOL Executive Function | -0.009 | 0.007 | -1.188 | 0.236 | -0.012 | 0.015 | -0.792 | 0.429 |
| Fluid Cognition | -0.003 | 0.005 | -0.676 | 0.500 | 0.006 | 0.006 | 1.019 | 0.309 |
| TOPF | -0.006 | 0.005 | -1.184 | 0.237 | -0.006 | 0.006 | -1.116 | 0.266 |
| AGE | -0.003 | 0.006 | -0.423 | 0.673 | 0.000 | 0.007 | -0.042 | 0.966 |
| PCL5 | 0.005 | 0.003 | 1.605 | 0.110 | 0.016 | 0.006 | 2.563 | 0.011 |
| PHQ9 | 0.007 | 0.010 | 0.628 | 0.530 | -0.044 | 0.020 | -2.179 | 0.030 |
| TBI Exposure | NA | NA | NA | NA | NA | NA | NA | NA |
| Unexposed | NA | NA | NA | NA | NA | NA | NA | NA |
| Combat mTBI | -0.045 | 0.159 | -0.280 | 0.780 | -0.118 | 0.216 | -0.547 | 0.585 |
| Non-Combat TBI | 0.085 | 0.175 | 0.487 | 0.627 | 0.006 | 0.188 | 0.034 | 0.973 |
| NUMBER OF BLAST PCES | -0.076 | 0.029 | -2.657 | 0.008 | -0.074 | 0.034 | -2.192 | 0.029 |
| Number of Positive mTBIs | -0.032 | 0.035 | -0.893 | 0.373 | -0.005 | 0.048 | -0.108 | 0.914 |
| Number of TBI with PTA+LOC | -0.040 | 0.105 | -0.384 | 0.701 | -0.137 | 0.120 | -1.140 | 0.255 |

1. **Temporal Left Alpha**

|  | Unadjusted | | | | Adjusted | | | |
| --- | --- | --- | --- | --- | --- | --- | --- | --- |
| Measure | Est. | SE | t | p | Est. | SE | t | p |
| DRRI2 | -0.003 | 0.004 | -0.755 | 0.451 | 0.004 | 0.006 | 0.737 | 0.462 |
| TBIQOL Cognition | 0.004 | 0.006 | 0.731 | 0.465 | 0.006 | 0.012 | 0.492 | 0.623 |
| TBIQOL Executive Function | -0.001 | 0.007 | -0.083 | 0.934 | -0.019 | 0.015 | -1.279 | 0.202 |
| Fluid Cognition | -0.001 | 0.005 | -0.110 | 0.913 | -0.001 | 0.006 | -0.165 | 0.869 |
| TOPF | 0.004 | 0.005 | 0.718 | 0.473 | 0.005 | 0.006 | 0.836 | 0.404 |
| AGE | 0.002 | 0.006 | 0.283 | 0.777 | -0.002 | 0.007 | -0.251 | 0.802 |
| PCL5 | -0.001 | 0.003 | -0.266 | 0.791 | 0.016 | 0.006 | 2.582 | 0.010 |
| PHQ9 | -0.014 | 0.009 | -1.464 | 0.144 | -0.064 | 0.020 | -3.203 | 0.002 |
| TBI Exposure | NA | NA | NA | NA | NA | NA | NA | NA |
| Unexposed | NA | NA | NA | NA | NA | NA | NA | NA |
| Combat mTBI | -0.216 | 0.143 | -1.512 | 0.131 | -0.417 | 0.212 | -1.970 | 0.050 |
| Non-Combat TBI | 0.031 | 0.157 | 0.201 | 0.841 | -0.052 | 0.185 | -0.281 | 0.779 |
| NUMBER OF BLAST PCES | -0.049 | 0.026 | -1.880 | 0.061 | -0.054 | 0.033 | -1.614 | 0.108 |
| Number of Positive mTBIs | -0.011 | 0.032 | -0.351 | 0.726 | 0.076 | 0.047 | 1.614 | 0.108 |
| Number of TBI with PTA+LOC | -0.143 | 0.094 | -1.516 | 0.131 | -0.232 | 0.118 | -1.974 | 0.049 |

1. **Temporal Left Beta**

|  | Unadjusted | | | | Adjusted | | | |
| --- | --- | --- | --- | --- | --- | --- | --- | --- |
| Measure | Est. | SE | t | p | Est. | SE | t | p |
| DRRI2 | -0.002 | 0.001 | -2.731 | 0.007 | -0.003 | 0.001 | -2.823 | 0.005 |
| TBIQOL Cognition | -0.001 | 0.001 | -0.998 | 0.319 | -0.006 | 0.002 | -2.840 | 0.005 |
| TBIQOL Executive Function | 0.000 | 0.001 | 0.230 | 0.818 | 0.004 | 0.003 | 1.514 | 0.131 |
| Fluid Cognition | 0.003 | 0.001 | 2.768 | 0.006 | 0.003 | 0.001 | 2.847 | 0.005 |
| TOPF | 0.001 | 0.001 | 1.167 | 0.244 | -0.001 | 0.001 | -0.527 | 0.598 |
| AGE | 0.001 | 0.001 | 0.532 | 0.595 | 0.002 | 0.001 | 1.621 | 0.106 |
| PCL5 | -0.001 | 0.001 | -1.547 | 0.123 | -0.001 | 0.001 | -1.053 | 0.293 |
| PHQ9 | -0.002 | 0.002 | -0.922 | 0.357 | 0.001 | 0.004 | 0.392 | 0.695 |
| TBI Exposure | NA | NA | NA | NA | NA | NA | NA | NA |
| Unexposed | NA | NA | NA | NA | NA | NA | NA | NA |
| Combat mTBI | -0.030 | 0.029 | -1.023 | 0.307 | -0.005 | 0.039 | -0.136 | 0.892 |
| Non-Combat TBI | -0.012 | 0.032 | -0.380 | 0.704 | -0.045 | 0.034 | -1.330 | 0.184 |
| NUMBER OF BLAST PCES | -0.004 | 0.005 | -0.790 | 0.430 | 0.001 | 0.006 | 0.142 | 0.887 |
| Number of Positive mTBIs | 0.004 | 0.006 | 0.642 | 0.521 | 0.024 | 0.009 | 2.767 | 0.006 |
| Number of TBI with PTA+LOC | -0.065 | 0.019 | -3.434 | 0.001 | -0.088 | 0.022 | -4.049 | 0.000 |

1. **Temporal Left Delta**

|  | Unadjusted | | | | Adjusted | | | |
| --- | --- | --- | --- | --- | --- | --- | --- | --- |
| Measure | Est. | SE | t | p | Est. | SE | t | p |
| DRRI2 | 0.011 | 0.005 | 2.203 | 0.028 | 0.000 | 0.007 | 0.067 | 0.947 |
| TBIQOL Cognition | -0.009 | 0.007 | -1.288 | 0.199 | 0.015 | 0.014 | 1.087 | 0.278 |
| TBIQOL Executive Function | -0.015 | 0.009 | -1.689 | 0.092 | -0.004 | 0.018 | -0.237 | 0.813 |
| Fluid Cognition | -0.011 | 0.006 | -1.826 | 0.069 | -0.007 | 0.007 | -0.970 | 0.333 |
| TOPF | -0.015 | 0.006 | -2.398 | 0.017 | -0.010 | 0.007 | -1.515 | 0.131 |
| AGE | -0.015 | 0.007 | -2.001 | 0.046 | -0.014 | 0.009 | -1.652 | 0.100 |
| PCL5 | 0.013 | 0.004 | 3.533 | 0.000 | 0.017 | 0.007 | 2.249 | 0.025 |
| PHQ9 | 0.024 | 0.012 | 2.038 | 0.042 | -0.010 | 0.024 | -0.428 | 0.669 |
| TBI Exposure | NA | NA | NA | NA | NA | NA | NA | NA |
| Unexposed | NA | NA | NA | NA | NA | NA | NA | NA |
| Combat mTBI | 0.228 | 0.180 | 1.263 | 0.207 | 0.113 | 0.254 | 0.446 | 0.656 |
| Non-Combat TBI | -0.098 | 0.198 | -0.494 | 0.622 | -0.122 | 0.221 | -0.550 | 0.583 |
| NUMBER OF BLAST PCES | 0.011 | 0.033 | 0.334 | 0.738 | -0.007 | 0.040 | -0.183 | 0.855 |
| Number of Positive mTBIs | 0.058 | 0.040 | 1.445 | 0.149 | 0.071 | 0.056 | 1.258 | 0.210 |
| Number of TBI with PTA+LOC | 0.087 | 0.119 | 0.728 | 0.467 | -0.116 | 0.141 | -0.822 | 0.412 |

1. **Temporal Left Gamma**

|  | Unadjusted | | | | Adjusted | | | |
| --- | --- | --- | --- | --- | --- | --- | --- | --- |
| Measure | Est. | SE | t | p | Est. | SE | t | p |
| DRRI2 | 0.000 | 0.000 | -0.346 | 0.730 | 0.000 | 0.000 | -1.879 | 0.061 |
| TBIQOL Cognition | 0.000 | 0.000 | -1.658 | 0.098 | -0.001 | 0.001 | -1.646 | 0.101 |
| TBIQOL Executive Function | 0.000 | 0.000 | -0.301 | 0.763 | 0.001 | 0.001 | 2.341 | 0.020 |
| Fluid Cognition | 0.000 | 0.000 | -0.873 | 0.383 | 0.000 | 0.000 | -0.961 | 0.337 |
| TOPF | 0.000 | 0.000 | 1.165 | 0.245 | 0.000 | 0.000 | 1.290 | 0.198 |
| AGE | 0.000 | 0.000 | 0.925 | 0.356 | 0.000 | 0.000 | 0.730 | 0.466 |
| PCL5 | 0.000 | 0.000 | 0.405 | 0.686 | 0.000 | 0.000 | -1.476 | 0.141 |
| PHQ9 | 0.001 | 0.000 | 1.380 | 0.169 | 0.002 | 0.001 | 2.397 | 0.017 |
| TBI Exposure | NA | NA | NA | NA | NA | NA | NA | NA |
| Unexposed | NA | NA | NA | NA | NA | NA | NA | NA |
| Combat mTBI | 0.011 | 0.006 | 1.698 | 0.090 | 0.013 | 0.009 | 1.432 | 0.153 |
| Non-Combat TBI | 0.007 | 0.007 | 1.014 | 0.311 | 0.000 | 0.008 | -0.005 | 0.996 |
| NUMBER OF BLAST PCES | 0.002 | 0.001 | 1.493 | 0.136 | 0.002 | 0.001 | 1.376 | 0.170 |
| Number of Positive mTBIs | 0.003 | 0.001 | 2.194 | 0.029 | 0.003 | 0.002 | 1.677 | 0.095 |
| Number of TBI with PTA+LOC | -0.005 | 0.004 | -1.169 | 0.243 | -0.013 | 0.005 | -2.549 | 0.011 |

1. **Temporal Left Theta**

|  | Unadjusted | | | | Adjusted | | | |
| --- | --- | --- | --- | --- | --- | --- | --- | --- |
| Measure | Est. | SE | t | p | Est. | SE | t | p |
| DRRI2 | 0.002 | 0.004 | 0.484 | 0.629 | 0.005 | 0.004 | 1.149 | 0.252 |
| TBIQOL Cognition | -0.003 | 0.004 | -0.623 | 0.534 | 0.001 | 0.008 | 0.132 | 0.895 |
| TBIQOL Executive Function | -0.003 | 0.005 | -0.622 | 0.535 | -0.013 | 0.011 | -1.215 | 0.225 |
| Fluid Cognition | -0.003 | 0.004 | -0.642 | 0.521 | 0.006 | 0.004 | 1.325 | 0.186 |
| TOPF | -0.004 | 0.005 | -0.930 | 0.353 | -0.005 | 0.004 | -1.245 | 0.214 |
| AGE | -0.002 | 0.005 | -0.325 | 0.745 | 0.002 | 0.005 | 0.418 | 0.676 |
| PCL5 | 0.000 | 0.003 | -0.019 | 0.985 | 0.006 | 0.004 | 1.425 | 0.155 |
| PHQ9 | -0.002 | 0.009 | -0.207 | 0.836 | -0.025 | 0.014 | -1.755 | 0.080 |
| TBI Exposure | NA | NA | NA | NA | NA | NA | NA | NA |
| Unexposed | NA | NA | NA | NA | NA | NA | NA | NA |
| Combat mTBI | -0.054 | 0.136 | -0.395 | 0.693 | -0.054 | 0.152 | -0.358 | 0.720 |
| Non-Combat TBI | 0.114 | 0.149 | 0.768 | 0.443 | -0.028 | 0.132 | -0.209 | 0.835 |
| NUMBER OF BLAST PCES | -0.053 | 0.025 | -2.142 | 0.033 | -0.037 | 0.024 | -1.571 | 0.117 |
| Number of Positive mTBIs | -0.029 | 0.030 | -0.963 | 0.336 | -0.003 | 0.034 | -0.097 | 0.922 |
| Number of TBI with PTA+LOC | -0.074 | 0.089 | -0.827 | 0.409 | -0.099 | 0.084 | -1.173 | 0.242 |

1. **Temporal Right Alpha**

|  | Unadjusted | | | | Adjusted | | | |
| --- | --- | --- | --- | --- | --- | --- | --- | --- |
| Measure | Est. | SE | t | p | Est. | SE | t | p |
| DRRI2 | -0.002 | 0.004 | -0.582 | 0.561 | 0.004 | 0.006 | 0.637 | 0.524 |
| TBIQOL Cognition | 0.005 | 0.006 | 0.897 | 0.371 | 0.009 | 0.013 | 0.712 | 0.477 |
| TBIQOL Executive Function | 0.000 | 0.008 | -0.005 | 0.996 | -0.023 | 0.016 | -1.444 | 0.150 |
| Fluid Cognition | -0.001 | 0.005 | -0.116 | 0.908 | -0.003 | 0.006 | -0.476 | 0.634 |
| TOPF | 0.007 | 0.005 | 1.254 | 0.211 | 0.008 | 0.006 | 1.360 | 0.175 |
| AGE | 0.001 | 0.006 | 0.227 | 0.820 | -0.003 | 0.008 | -0.414 | 0.679 |
| PCL5 | -0.001 | 0.003 | -0.417 | 0.677 | 0.017 | 0.007 | 2.659 | 0.008 |
| PHQ9 | -0.017 | 0.010 | -1.665 | 0.097 | -0.071 | 0.021 | -3.338 | 0.001 |
| TBI Exposure | NA | NA | NA | NA | NA | NA | NA | NA |
| Unexposed | NA | NA | NA | NA | NA | NA | NA | NA |
| Combat mTBI | -0.302 | 0.153 | -1.975 | 0.049 | -0.603 | 0.225 | -2.677 | 0.008 |
| Non-Combat TBI | -0.076 | 0.168 | -0.455 | 0.650 | -0.204 | 0.196 | -1.041 | 0.299 |
| NUMBER OF BLAST PCES | -0.030 | 0.028 | -1.080 | 0.281 | -0.034 | 0.035 | -0.961 | 0.337 |
| Number of Positive mTBIs | -0.003 | 0.034 | -0.095 | 0.924 | 0.102 | 0.050 | 2.042 | 0.042 |
| Number of TBI with PTA+LOC | -0.158 | 0.101 | -1.566 | 0.118 | -0.264 | 0.125 | -2.113 | 0.035 |

|  |  |  |  |  |  |  |  |  |
| --- | --- | --- | --- | --- | --- | --- | --- | --- |

1. **Temporal Right Beta**

|  | Unadjusted | | | | Adjusted | | | |
| --- | --- | --- | --- | --- | --- | --- | --- | --- |
| Measure | Est. | SE | t | p | Est. | SE | t | p |
| DRRI2 | -0.002 | 0.001 | -2.747 | 0.006 | -0.002 | 0.001 | -2.057 | 0.041 |
| TBIQOL Cognition | 0.000 | 0.001 | -0.394 | 0.693 | -0.003 | 0.002 | -1.505 | 0.133 |
| TBIQOL Executive Function | 0.000 | 0.001 | 0.098 | 0.922 | 0.002 | 0.003 | 0.775 | 0.439 |
| Fluid Cognition | 0.002 | 0.001 | 1.890 | 0.060 | 0.003 | 0.001 | 2.471 | 0.014 |
| TOPF | 0.001 | 0.001 | 0.602 | 0.548 | 0.000 | 0.001 | -0.430 | 0.668 |
| AGE | 0.001 | 0.001 | 1.191 | 0.234 | 0.002 | 0.001 | 1.730 | 0.085 |
| PCL5 | 0.000 | 0.001 | -0.427 | 0.669 | 0.000 | 0.001 | 0.408 | 0.683 |
| PHQ9 | -0.002 | 0.002 | -0.790 | 0.430 | -0.002 | 0.004 | -0.609 | 0.543 |
| TBI Exposure | NA | NA | NA | NA | NA | NA | NA | NA |
| Unexposed | NA | NA | NA | NA | NA | NA | NA | NA |
| Combat mTBI | -0.031 | 0.029 | -1.041 | 0.299 | -0.034 | 0.039 | -0.862 | 0.390 |
| Non-Combat TBI | 0.005 | 0.032 | 0.165 | 0.869 | -0.034 | 0.034 | -1.001 | 0.318 |
| NUMBER OF BLAST PCES | -0.007 | 0.005 | -1.344 | 0.180 | -0.003 | 0.006 | -0.405 | 0.686 |
| Number of Positive mTBIs | 0.006 | 0.007 | 0.907 | 0.365 | 0.027 | 0.009 | 3.103 | 0.002 |
| Number of TBI with PTA+LOC | -0.049 | 0.019 | -2.549 | 0.011 | -0.068 | 0.022 | -3.115 | 0.002 |

1. **Temporal Right Delta**

|  | Unadjusted | | | | Adjusted | | | |
| --- | --- | --- | --- | --- | --- | --- | --- | --- |
| Measure | Est. | SE | t | p | Est. | SE | t | p |
| DRRI2 | 0.006 | 0.006 | 1.029 | 0.304 | -0.003 | 0.007 | -0.335 | 0.738 |
| TBIQOL Cognition | -0.011 | 0.008 | -1.332 | 0.184 | 0.014 | 0.016 | 0.897 | 0.370 |
| TBIQOL Executive Function | -0.019 | 0.010 | -2.004 | 0.046 | -0.007 | 0.019 | -0.376 | 0.707 |
| Fluid Cognition | -0.020 | 0.007 | -3.102 | 0.002 | -0.015 | 0.008 | -1.943 | 0.053 |
| TOPF | -0.020 | 0.007 | -2.836 | 0.005 | -0.010 | 0.007 | -1.368 | 0.172 |
| AGE | -0.007 | 0.008 | -0.863 | 0.389 | -0.012 | 0.009 | -1.292 | 0.197 |
| PCL5 | 0.016 | 0.004 | 3.816 | 0.000 | 0.019 | 0.008 | 2.358 | 0.019 |
| PHQ9 | 0.028 | 0.013 | 2.124 | 0.034 | -0.023 | 0.027 | -0.868 | 0.386 |
| TBI Exposure | NA | NA | NA | NA | NA | NA | NA | NA |
| Unexposed | NA | NA | NA | NA | NA | NA | NA | NA |
| Combat mTBI | 0.045 | 0.204 | 0.220 | 0.826 | 0.121 | 0.281 | 0.432 | 0.666 |
| Non-Combat TBI | -0.147 | 0.224 | -0.657 | 0.512 | 0.018 | 0.245 | 0.074 | 0.941 |
| NUMBER OF BLAST PCES | -0.007 | 0.037 | -0.178 | 0.859 | 0.021 | 0.044 | 0.471 | 0.638 |
| Number of Positive mTBIs | -0.002 | 0.045 | -0.045 | 0.964 | -0.036 | 0.062 | -0.585 | 0.559 |
| Number of TBI with PTA+LOC | 0.149 | 0.134 | 1.110 | 0.268 | 0.052 | 0.156 | 0.334 | 0.739 |

1. **Temporal Right Gamma**

|  | Unadjusted | | | | Adjusted | | | |
| --- | --- | --- | --- | --- | --- | --- | --- | --- |
| Measure | Est. | SE | t | p | Est. | SE | t | p |
| DRRI2 | 0.000 | 0.000 | -0.262 | 0.794 | 0.000 | 0.000 | -0.755 | 0.451 |
| TBIQOL Cognition | 0.000 | 0.000 | -0.991 | 0.322 | 0.000 | 0.000 | -0.145 | 0.885 |
| TBIQOL Executive Function | 0.000 | 0.000 | -0.534 | 0.593 | 0.001 | 0.001 | 1.124 | 0.262 |
| Fluid Cognition | 0.000 | 0.000 | -0.968 | 0.334 | 0.000 | 0.000 | -0.501 | 0.617 |
| TOPF | 0.000 | 0.000 | 0.494 | 0.622 | 0.000 | 0.000 | 0.922 | 0.357 |
| AGE | 0.000 | 0.000 | 1.432 | 0.153 | 0.000 | 0.000 | 1.050 | 0.295 |
| PCL5 | 0.000 | 0.000 | 1.129 | 0.260 | 0.000 | 0.000 | -0.101 | 0.920 |
| PHQ9 | 0.000 | 0.000 | 1.171 | 0.243 | 0.001 | 0.001 | 1.173 | 0.242 |
| TBI Exposure | NA | NA | NA | NA | NA | NA | NA | NA |
| Unexposed | NA | NA | NA | NA | NA | NA | NA | NA |
| Combat mTBI | 0.009 | 0.006 | 1.467 | 0.143 | 0.004 | 0.009 | 0.459 | 0.647 |
| Non-Combat TBI | 0.011 | 0.007 | 1.664 | 0.097 | 0.003 | 0.008 | 0.338 | 0.735 |
| NUMBER OF BLAST PCES | 0.001 | 0.001 | 0.866 | 0.387 | 0.001 | 0.001 | 0.742 | 0.459 |
| Number of Positive mTBIs | 0.004 | 0.001 | 2.656 | 0.008 | 0.005 | 0.002 | 2.525 | 0.012 |
| Number of TBI with PTA+LOC | -0.003 | 0.004 | -0.671 | 0.503 | -0.010 | 0.005 | -2.110 | 0.036 |

1. **Temporal Right Theta**

|  | Unadjusted | | | | Adjusted | | | |
| --- | --- | --- | --- | --- | --- | --- | --- | --- |
| Measure | Est. | SE | t | p | Est. | SE | t | p |
| DRRI2 | 0.002 | 0.003 | 0.755 | 0.451 | 0.004 | 0.004 | 1.054 | 0.293 |
| TBIQOL Cognition | -0.003 | 0.004 | -0.744 | 0.457 | 0.002 | 0.008 | 0.254 | 0.800 |
| TBIQOL Executive Function | -0.005 | 0.005 | -0.886 | 0.376 | -0.013 | 0.010 | -1.293 | 0.197 |
| Fluid Cognition | -0.004 | 0.004 | -0.972 | 0.332 | 0.003 | 0.004 | 0.639 | 0.523 |
| TOPF | -0.004 | 0.004 | -1.000 | 0.318 | -0.004 | 0.004 | -1.028 | 0.305 |
| AGE | -0.001 | 0.005 | -0.295 | 0.768 | 0.001 | 0.005 | 0.168 | 0.866 |
| PCL5 | 0.002 | 0.002 | 0.740 | 0.460 | 0.009 | 0.004 | 2.044 | 0.042 |
| PHQ9 | 0.000 | 0.008 | -0.022 | 0.983 | -0.031 | 0.014 | -2.221 | 0.027 |
| TBI Exposure | NA | NA | NA | NA | NA | NA | NA | NA |
| Unexposed | NA | NA | NA | NA | NA | NA | NA | NA |
| Combat mTBI | -0.077 | 0.117 | -0.657 | 0.512 | -0.100 | 0.150 | -0.665 | 0.506 |
| Non-Combat TBI | 0.047 | 0.129 | 0.365 | 0.716 | -0.035 | 0.131 | -0.272 | 0.786 |
| NUMBER OF BLAST PCES | -0.042 | 0.021 | -1.970 | 0.050 | -0.028 | 0.023 | -1.193 | 0.234 |
| Number of Positive mTBIs | -0.032 | 0.026 | -1.245 | 0.214 | -0.018 | 0.033 | -0.534 | 0.594 |
| Number of TBI with PTA+LOC | -0.038 | 0.077 | -0.495 | 0.621 | -0.056 | 0.083 | -0.673 | 0.501 |
